# Preoperative low-energy diets for patients with a body mass index >30 kg/m^2^ undergoing non-bariatric surgery: pilot feasibility randomized clinical trial and a systematic review and meta-analysis of efficacy data

**DOI:** 10.1093/bjs/znag023

**Published:** 2026-03-13

**Authors:** Tyler McKechnie, Olivia Kuszaj, Heather Perks, Sahaar Rattansi, Carolina Meyhofer Pedroso, Phillip Staibano, Alex Thabane, Jordan Leitch, Deborah DuMerton, Sally Griffin, Dimitrios A Koutoukidis, Karim Ramji, Sunil V Patel, Aristithes Doumouras, Cagla Eskicioglu, Sameer Parpia, Lehana Thabane, Mohit Bhandari

**Affiliations:** Division of General Surgery, Department of Surgery, McMaster University, Hamilton, Ontario, Canada; Department of Health Research Methods, Evidence, and Impact, McMaster University, Hamilton, Ontario, Canada; Division of General Surgery, Department of Surgery, McMaster University, Hamilton, Ontario, Canada; Division of General Surgery, Department of Surgery, McMaster University, Hamilton, Ontario, Canada; Division of General Surgery, Department of Surgery, McMaster University, Hamilton, Ontario, Canada; Division of General Surgery, Department of Surgery, McMaster University, Hamilton, Ontario, Canada; Department of Health Research Methods, Evidence, and Impact, McMaster University, Hamilton, Ontario, Canada; Division of Otolaryngology – Head and Neck Surgery, Department of Surgery, McMaster University, Hamilton, Ontario, Canada; Department of Health Research Methods, Evidence, and Impact, McMaster University, Hamilton, Ontario, Canada; Department of Anesthesia and Perioperative Medicine, Queen’s University, Kingston Health Sciences Centre, Kingston, Ontario, Canada; Department of Anesthesia and Perioperative Medicine, Queen’s University, Kingston Health Sciences Centre, Kingston, Ontario, Canada; School of Exercise and Nutrition Sciences, Queensland University of Technology, Brisbane, Queensland, Australia; Nuffield Department of Primary Care Health Sciences, University of Oxford, Oxford, UK; Division of General Surgery, Department of Surgery, McMaster University, Hamilton, Ontario, Canada; Division of General Surgery, Department of Surgery, St. Joseph Healthcare Hamilton, Hamilton, Ontario, Canada; Division of General Surgery, Department of Surgery, Queen’s University, Kingston Health Sciences Centre, Kingston, Ontario, Canada; Division of General Surgery, Department of Surgery, McMaster University, Hamilton, Ontario, Canada; Department of Health Research Methods, Evidence, and Impact, McMaster University, Hamilton, Ontario, Canada; Division of General Surgery, Department of Surgery, St. Joseph Healthcare Hamilton, Hamilton, Ontario, Canada; Division of General Surgery, Department of Surgery, McMaster University, Hamilton, Ontario, Canada; Division of General Surgery, Department of Surgery, St. Joseph Healthcare Hamilton, Hamilton, Ontario, Canada; Department of Health Research Methods, Evidence, and Impact, McMaster University, Hamilton, Ontario, Canada; Department of Oncology, McMaster University, Hamilton, Ontario, Canada; Research Institute of St. Joe’s Hamilton, St. Joseph’s Healthcare Hamilton, Hamilton, Ontario, Canada; Faculty of Health Sciences, University of Johannesburg, Johannesburg, South Africa; Department of Health Research Methods, Evidence, and Impact, McMaster University, Hamilton, Ontario, Canada; Division of Orthopedic Surgery, Department of Surgery, McMaster University, Hamilton, Ontario, Canada

## Abstract

**Background:**

The current evidence for preoperative low-energy diets (LEDs) for patients with a BMI >30 kg/m^2^ before non-bariatric surgery rests on studies with a high risk of bias. An RCT is warranted to bridge this knowledge gap. Before a larger RCT, a pilot feasibility RCT was conducted to address potential hurdles for the larger trial.

**Methods:**

The pilot feasibility multicentre trial was conducted in Canada at four centres between 3 January 2024 and 23 October 2024. Patients were randomized (1 : 1 randomly permuted online blocked allocation) to receive a 3-week LED protocol or standard care. All patients aged >18 years with a BMI >30 kg/m^2^ undergoing elective non-bariatric intra-abdominal or orthopaedic surgery were evaluated for enrolment. Main exclusion criteria were LED contraindications and surgery scheduled without at least 3 weeks notice. The primary outcome was descriptive including the following feasibility outcomes: recruitment rate, randomization percentage, intervention adherence, and follow-up completion. Clinical outcomes included anthropometric data. The primary outcome analysis was descriptive. Additionally, a random-effects meta-analysis was performed using data from the present study and data from previously published RCTs to evaluate 30-day postoperative morbidity. The last follow-up date was 14 January 2025.

**Results:**

Out of 373 patients approached, 288 were eligible. Of these, 175 consented to be randomized (60.8% (95% c.i. 54.9–66.4%)). Seventy four of these patients were still awaiting a surgical date when the trial closed, leaving 91 patients randomized (LED *n* = 45, control *n* = 46) in the present pilot trial. Out of these 91 randomized patients, 81 (89%, 95% c.i. 80.7–94.6%) had complete follow-up. LED adherence was 81.7% (95% c.i. 74.1–89.3%). Analysis of covariance suggested patients in the LED group lost more weight during the 3-week intervention period (MD 4.5kg, 95% c.i. −5.6 to −3.5). The pooled meta-analysis of 5 RCTs suggested a 19% relative risk reduction in postoperative morbidity favouring the intervention (RR 0.81, 95% c.i. 0.52–1.26, *P* = 0.351, *I*^2^ = 0%).

**Conclusion:**

The feasibility targets of this pilot RCT were not met for recruitment rate, randomization percentage, and complete follow-up. The pooled meta-analysis suggests that LEDs before non-bariatric surgery can effectively induce weight loss with reduced postoperative morbidity, which needs to be validated in a larger non-inferiority RCT with additional centres to meet the feasibility targets.

**Registration number:**

NCT03935451 (http://www.clinicaltrials.gov).

## Teaser

A pilot RCT was conducted to evaluate the feasibility of a definitive trial for preoperative low-energy diets before non-bariatric surgery. Not all of the feasibility targets were met, in particular recruitment rate and randomization percentage. Feasibility limitations can be addressed by adding more sites and a definitive trial should be considered.

Key messages

- Current evidence evaluating the use of preoperative low-energy diets (LEDs) for optimizing non-bariatric surgery outcomes is scarce.
- A pilot feasibility RCT was conducted to assess the possibility of a larger non-inferiority RCT for this intervention.
- Not all of the feasibility targets were met, in particular recruitment rate and randomization percentage.
- A meta-analysis was performed using data from the present study and data from previously published RCTs for the primary efficacy outcome, suggesting there may be important benefits associated with preoperative LEDs.
- Feasibility limitations can be addressed by adding more sites and a definitive trial should be considered.

## Introduction

Clinical obesity is increasingly prevalent in Western society and around the world^1^. Over 90 million individuals currently live with a BMI >30 kg/m^2^ in the USA alone—a number that is expected to rise to over half of the population by 2030^2^. With 18% of individuals aged 5-14 years and 20% of individuals aged 15-24 years living with being overweight or obese as of 2021, this problem is only expected to worsen^1^. As such, the surgical patient living with excess weight is unavoidable.

Patients living with obesity are at particularly higher risk of intraoperative and postoperative complications^3,4^. Practically, excess intra-abdominal adiposity is associated with increased technical difficulty intraoperatively for surgeons, especially when working within the confines of certain surgical spaces such as the upper gastrointestinal tract, pelvis, and joints^5–7^. In metabolic bariatric surgery (MBS), patients undergo a multidisciplinary process before surgery to support preoperative weight loss and the incorporation of permanent life-style changes to optimize MBS outcomes. Specifically with regards to preoperative weight loss, these patients adhere to very low-energy diets (VLEDs) or low-energy diets (LEDs)^8,9^. VLEDs/LEDs are an intensive approach to short-term medical weight loss that include all recommended daily micronutrients and are high in protein, yet are limited in their fat and carbohydrate content, allowing them to promote weight loss while maintaining lean body mass^10–12^. These programmes are safe, are tolerable, and have been associated with decreased fat volume, surgeon-perceived operative difficulty, operating time, postoperative length of stay (LOS), and postoperative morbidity (relative risk 0.67 (95% c.i. 0.39 to 1.17)) for patients undergoing MBS^13–15^. While the most recent international consensus position from the International Federation for the Surgery and Other Therapies for Obesity (IFSO) states there is insufficient evidence to routinely recommend preoperative obesity management medications (OMMs) before MBS, these medications are also associated with significant preoperative weight loss in this population^16,17^. Given the associated advantages in the MBS population, it follows that a similar LED programme could have clinical utility for patients living with excess weight undergoing non-bariatric surgery.

A recent systematic review and meta-analysis of 13 studies evaluating the use of preoperative VLEDs/LEDs for patients with a BMI >30 kg/m^2^ undergoing non-MBS (that is orthopaedic surgery, vascular surgery, colorectal surgery, upper gastrointestinal surgery, gynaecological surgery, and a variety of general surgery procedures) found that preoperative VLEDs/LEDs reliably resulted in significant weight loss (3.2–19.2 kg), with high adherence rates (94–100%) and a low risk of adverse events (<14% in most studies)^18^. However, previous studies were small, heterogeneous, and of variable methodological quality. Moreover, a national survey of 78 surgeons performing major non-bariatric surgery identified a disparity between the percentage of surgeons routinely prescribing VLEDs/LEDs for preoperative weight loss before elective non-bariatric surgery (30%) and the percentage who would be willing to routinely prescribe preoperative VLEDs/LEDs with more familiarity and evidence (80%)^19^. Altogether, these data warrant a large RCT to confidently support LED use. The aim of this pilot feasibility multicentre RCT was to compare the efficacy of LEDs with that of standard care in terms of perioperative outcomes for patients with obesity (with a BMI >30 kg/m^2^) undergoing major non-bariatric surgery, assessing the following critical feasibility outcomes: recruitment ability; adherence to LED regimens; and ability to completely follow-up patients.

## Methods

### Trial design

A 1 : 1 parallel multicentre pilot RCT was conducted, with surgeons, outcome assessors, and data analysts all blinded. The study adhered to the CONSORT extension for randomized pilot and feasibility trials (*Appendices S1*, *S2*)^20^. The trial protocol was published in *Pilot and Feasibility Studies* in May 2024 (*File S1*)^21^. Amendments to this protocol included allowing the inclusion of patients undergoing spinal anaesthetic for orthopaedic surgery as well as potential truncation of the LED protocol by a maximum of 2 days at the start of the protocol to allow for flexibility in lieu of logistical constraints surrounding recruitment. Additionally, a preoperative change in grip strength was added as a balancing outcome measure to assess for deleterious effects associated with LED use, as preoperative grip strength is a surrogate for lean body mass^22^. The terminology was updated from VLEDs to LEDs as the intervention contained 920 kcal/day, more than the strict definition of <800 kcal/day for VLEDs^23^. Lastly, a meta-analysis of the main clinical outcome integral to the conduct of a potential definitive trial, overall 30-day postoperative morbidity, was conducted. Local ethics board approval was obtained from the Hamilton Integrated Research Ethics Board (Project #15946) and the Queen’s University Health Sciences and Affiliated Teaching Hospitals Research Ethics Board (Project #6040111). The trial was registered on ClinicalTrials.gov (NCT03935451).

### Study participants

Patients were recruited across four separate centres: St. Joseph’s Healthcare Hamilton; Juravinski Hospital, Hamilton; Hamilton General Hospital; and Kingston Health Sciences Centre. Patients aged >18 years with a BMI >30 kg/m^2^ undergoing major elective non-bariatric surgery were included. Major surgery was defined as any abdominal or orthopaedic operation performed under general anaesthesia or spinal anaesthesia requiring a skin incision extending beyond the subcutaneous tissue. Patients with recently diagnosed myocardial infarction or unstable angina (within the past 6 months), moderate-to-severe renal dysfunction (estimated glomerular filtration rate <30 ml/min/1.73 m^2^), severe liver dysfunction (cirrhosis, portal hypertension, hepatic encephalopathy, or hepatorenal syndrome), recently diagnosed alcohol- or drug-use disorders (excessive use of the substance within the past 6 months), recently diagnosed uncontrolled eating disorders (such as bulimia nervosa or binge-eating disorder within the past 12 months), a recent episode of gout (within the past 6 months), a history of porphyria, and/or a known allergy to LED ingredients were excluded. Pregnant and/or breastfeeding women were excluded. Lastly, patients residing in long-term care facilities and patients unable to provide written informed consent were not eligible. Co-enrolment was evaluated on a case-by-case basis by the steering committee.

### Randomization

Patients meeting inclusion/exclusion criteria were approached by designated research personnel at the time of their initial surgical consultation. Patients consenting to participate in the trial were registered in the trial database. Once a surgery date was obtained for consented patients who were on a waiting list awaiting a surgical date, permitting the surgery date was given at least 21 days in advance, patients were then allocated in a 1 : 1 ratio according to a computer-generated randomization schedule utilizing randomly permuted block sizes of four, six, or eight. Randomization was stratified according to study centre. Due to the nature of the intervention, patients could not be blinded. Allocation was concealed from patients until their initial pre-intervention visit was completed. Surgeons, outcome assessors, and statisticians were blinded to allocation.

### Trial interventions

All patients in the intervention group received surgeon-led counselling on weight loss and a 3-week LED protocol using Medimeal^®^ (Minogue Medical Inc.^©^, Saint-Laurent, Quebec, Canada). The LED protocol was adapted from the protocol of a local MBS centre of excellence. The LED was provided free of charge to participants. The intervention interval commenced 23 days before the scheduled date of surgery and concluded on the evening before the day prior to surgery. Patients consumed four packets of powdered Medimeal^®^ mixed with water per day, providing a total of 920 kcal/day. Additionally, patients were allowed to consume up to two cups of non-starchy vegetables per day and ad-lib zero-calorie fluids. Patients were provided with handouts with details pertaining to the prescribed diet at the time of their pre-intervention visit (*Appendix S3*) as well as diet diaries to track oral intake during the intervention interval. Patients living with diabetes who were randomized to the intervention arm were referred to a diabetes nurse educator by an unblinded research assistant for anti-hyperglycaemic management during the intervention interval.

Patients randomized to the control group received preoperative standard care, which consisted of surgeon-led preoperative weight loss counselling alone. There was no specific counselling recommended to surgeons to increase the pragmaticism of the trial. Patients did not receive prescriptions for any other weight loss supplement or any physical activity intervention aimed at promoting weight loss before surgery. Patients in the control group also tracked oral intake via diet diaries during the 3-week intervention interval.

All other aspects of perioperative care for patients in either arm were left to the discretion of the treating surgeon. All patients were enrolled in enhanced recovery after surgery perioperative programmes according to the type of operative intervention they were undergoing.

### Data collection

Patients were seen in person before commencement of the intervention interval (that is 23–30 days before surgery), on the day of their surgery (that is at the post-intervention visit), and approximately 30 days after surgery. Data regarding baseline patient demographics, disease characteristics, treatment characteristics, LED-associated adverse events, and postoperative clinical outcomes were a combination of patient self-reported data and electronic medical record data. Anthropometric measurements were performed at each in-person visit by a research assistant blinded to allocation. Quality-of-life data were obtained via administration of the Short-Form 36 (SF-36) questionnaire by a blinded research assistant at the pre-intervention, post-intervention, and postoperative follow-up visits. Adherence data were collected via the patient-reported diet diaries that were collected at the post-intervention visit. The number of patients who were approached, the number of patients who consented, and the number of patients who declined as well as the reasons why patients declined were recorded to assess feasibility. The Standard Protocol Items: Recommendations for Interventional Trials (SPIRIT) figure for participant timeline and data collection is reported in *Appendix S4*^24^.

Data for the meta-analysis were collected from the authors’ previously conducted systematic review and meta-analysis of preoperative VLEDs before non-bariatric surgery. Only RCT data were included. Additionally, an updated search of the literature up to 13 June 2025 was performed, to identify any subsequently published RCTs comparing VLEDs/LEDs with control for patients undergoing elective non-bariatric surgery. The search strategy is reported in *Appendix S5*.

### Feasibility outcomes

The feasibility outcomes included recruitment, randomization, intervention adherence, follow-up completion, and network development. Detailed definitions for each feasibility outcome are reported in the published study protocol^21^. The feasibility outcomes were assessed according to traffic light criteria: green—supports feasibility of a definitive trial; yellow—may support feasibility of a definitive trial with modifications; and red—does not support feasibility of a definitive trial^25^. The green, yellow, and red light criteria for each of the feasibility outcomes are reported in *Table S1*.

### Other outcomes

Safety outcomes were assessed by recording adverse events secondary to the use of preoperative LEDs in an analogous fashion to the OPTIWIN study; the largest medical weight loss RCT evaluating LEDs^26^. Minor adverse events included: constipation, diarrhoea, nausea, fatigue, dizziness, headache, and alopecia. Major adverse events included: acute kidney injury, electrolyte disturbances, cardiac arrhythmias, symptomatic gallstones, pancreatitis, pyelonephritis, and gout.

Clinical outcomes included anthropometric data; specifically, preoperative weight change, preoperative BMI change, preoperative waist circumference change, and preoperative grip strength change. Other clinical endpoints included overall 30-day postoperative morbidity, 30-day postoperative mortality, postoperative LOS, operating time, estimated intraoperative blood loss, intraoperative complications, and patient-reported quality of life (according to the SF-36 general health score). Thirty-day postoperative morbidity was the proposed primary efficacy endpoint for a definitive trial. Detailed definitions for each safety and clinical outcome are reported in the published study protocol^21^. Clinical efficacy outcomes were exploratory in nature given the pilot trial design.

### Sample size calculation

The sample size for this pilot RCT was 88 patients, estimated using a 95% confidence interval precision approach for the follow-up completion outcome. To assess a feasibility target of 90% follow-up completion, 88 patients would provide a 95% confidence interval of 82–95%, which would fall above the red light-criteria for feasibility (that is, not feasible).

### Statistical analyses

Descriptive statistics were used to characterize the sample population. Continuous variables are presented as mean(s.d.) or median (interquartile range (i.q.r.)), whereas categorical variables are presented as *n* (%). The primary outcome analysis was descriptive in nature and focused on feasibility. Differences in binary outcomes are presented as absolute risk differences (RDs) with 95% confidence intervals. Differences in continuous outcomes are presented as mean differences (MDs) with 95% confidence intervals. Analysis of covariance (ANCOVA) was used to calculate MDs for postintervention and postoperative continuous data, controlling for baseline anthropometric measurements. Hypothesis testing for outcomes was not conducted, in accordance with the CONSORT extension for randomized pilot and feasibility trials^20^. A DerSimonian–Laird random-effects meta-analysis for 30-day postoperative morbidity was performed. Pooled effect estimates were obtained by calculating the risk ratios (RRs) along with their respective 95% confidence intervals. All analyses were performed using STATA version 18 (StataCorp, College, TX, USA) by a member of the research team blinded to allocation.

## Results

### Participant recruitment

**Fig. 1:**
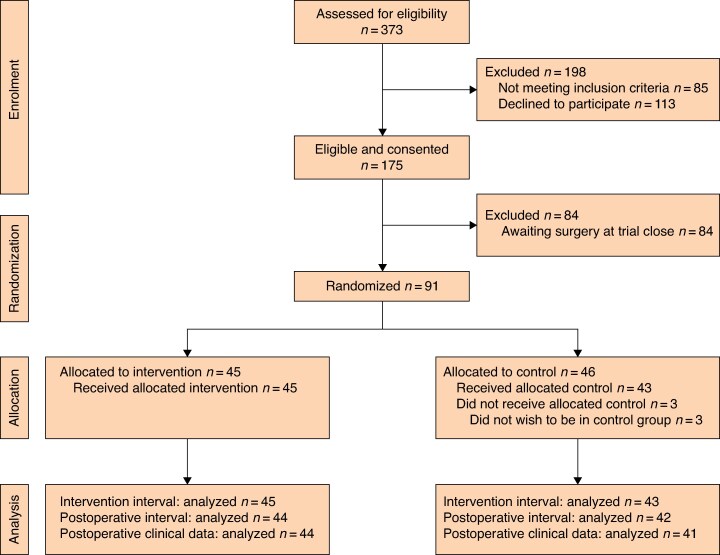
CONSORT flow diagram

The CONSORT flow diagram is presented in *Fig. 1*. Between 3 January 2024 and 23 October 2024, 373 patients were assessed for inclusion in the trial. A total of 85 patients were excluded based on inclusion/exclusion criteria and 113 patients declined to participate. The most common reasons for not wanting to participate included reluctance to come in for extra visits before surgery (21 of 62 (33.3%)) and feeling too overwhelmed by the idea of surgery to participate (19 of 62 (30.7%)) (*Table S2*). Ultimately, 175 patients were eligible and consented to participate, but the trial was closed (that is, reached sample size) before 84 of these patients had a scheduled surgical date, leaving 91 of these patients to be randomized. This was inflated by three patients beyond the a priori sample size to account for three post-randomization dropouts that occurred during the conduct of the trial.

### Baseline characteristics

Baseline patient characteristics are reported in *Table 1*. There were 45 patients randomized to the LED group and 46 patients randomized to the control group. There was no evidence of significant differences in mean baseline BMIs and waist circumferences between groups. Baseline grip strength was higher in the LED group. There were more active smokers and more patients with a history of previous surgery in the same surgical field in the control group.

**Table 1:** Baseline patient characteristics

Characteristic	LED group (*n* = 45)	Control group (*n* = 46)
Age (years), mean(s.d.)	58.6(11.3)	55.1(12.9)
Female	21 (46.7)	30 (65.2)
BMI (kg/m^2^), mean(s.d.)	36.1(5.6)	36.4(6.4)
Weight (kg), mean(s.d.)	107.8(21.5)	105.7(18.4)
Waist circumference (cm), mean(s.d.)	118.5(13.4)	116.3(12.6)
Grip strength (kg), mean(s.d.)	30.3(12.0)	25.1(9.1)
**Race**		
White	40 (88.9)	43 (93.5)
Black	2 (4.4)	1 (2.2)
Latin American	2 (4.4)	1 (2.2)
Arab	1 (2.2)	1 (2.2)
**Ethnicity**		
North American origin	36 (80.0)	32 (69.6)
European origin	7 (15.6)	12 (26.1)
Latin, Central, or South American origin	2 (4.4)	1 (2.2)
Asian origin	0 (0.0)	1 (2.2)
Smoker	4 (8.9)	9 (19.6)
CCI, median (i.q.r.)	2 (1–3)	2 (0–4)
mFI-11, median (i.q.r.)	0 (0–1)	1 (0–1)
ASA grade, median (i.q.r.)	III (III–III)	III (III–IV)
Diabetes	7 (15.6)	8 (17.4)
History of previous surgery in the same surgical field	18 (40.0)	23 (50.0)

Values are *n* (%) unless otherwise indicated. LED, low-energy diet; CCI, Charlson co-morbidity index; i.q.r., interquartile range; mFI-11, modified frailty index-11.

### Disease and treatment characteristics

Detailed disease and treatment characteristics are reported in *Table 2*. The indications for surgery were heterogeneous. More patients in the LED group underwent surgery for abdominal wall hernias and osteoarthritis, whereas more patients in the control group underwent surgery for colorectal neoplasia and benign biliary disease. In addition, more patients in the LED group underwent total knee arthroplasty and hernia repair with mesh, while more patients in the control group underwent proctectomy and cholecystectomy.

**Table 2:** Disease and treatment characteristics

Characteristic	LED group (*n* = 45)	Control group (*n* = 46)
**Disease characteristics**		
Disease		
Colorectal neoplasia	5 (11.1)	11 (23.9)
Diverticular disease	1 (2.2)	2 (4.3)
Stoma status	2 (4.4)	1 (2.2)
Rectal prolapse	1 (2.2)	0 (0.0)
Benign biliary disease	11 (24.4)	14 (30.4)
Abdominal wall hernia	10 (22.2)	8 (17.4)
Hiatal hernia	0 (0.0)	1 (2.2)
GIST	1 (2.2)	2 (4.3)
NET	1 (2.2)	0 (0.0)
Endometrial cancer	1 (2.2)	0 (0.0)
Osteoarthritis	12 (26.7)	7 (15.2)
Malignant disease	7 (15.6)	13 (28.3)
**Treatment characteristics**		
Procedure		
Colectomy	5 (11.1)	5 (10.9)
Proctectomy	2 (4.4)	5 (10.9)
Ileostomy reversal	0 (0.0)	1 (2.2)
Colostomy reversal	2 (4.4)	0 (0.0)
Gastrectomy	1 (2.2)	1 (2.2)
Pancreaticoduodenectomy	0 (0.0)	1 (2.2)
Hepatectomy	1 (2.2)	3 (6.5)
Cholecystectomy	11 (24.4)	14 (30.4)
Hysterectomy	1 (2.2)	0 (0.0)
Hernia repair with mesh	8 (17.8)	5 (10.9)
Hernia repair without mesh	2 (4.4)	3 (6.5)
Hiatal hernia repair	0 (0.0)	1 (2.2)
THA	3 (6.7)	3 (6.5)
TKA	9 (20.0)	4 (8.7)
MIS*, *n* of *n* (%)	24 of 33 (72.7)	30 of 39 (76.9)
MIS converted to open*, *n* of *n* (%)	1 of 33 (4.2)	0 of 39 (0.0)
Spinal anaesthetic	13 (29.5)	4 (9.8)

Values are *n* (%) unless otherwise indicated. *Intra-abdominal surgery only. LED, low-energy diet; GIST, gastrointestinal stromal tumour; NET, neuroendocrine tumour; THA, total hip arthroplasty; TKA, total knee arthroplasty; MIS, minimally invasive surgery.

### Feasibility outcomes

The feasibility outcomes, corresponding success criteria, and interpretations are reported in *Table 3*. The recruitment rate was 9.4 patients per month (91 patients randomized over 9.67 months), which does not support the feasibility of a definitive trial.

**Table 3:** Feasibility outcomes, corresponding success criteria, and interpretations

Feasibility outcome	Definition of success	Feasibility data	Interpretation
Recruitment rate: number of patients randomized per month	16 patients per month	9.4 patients recruited per month	Not feasible (red)
Randomization percentage: number of patients agreeing to be randomized divided by the number of eligible patients approached	≥70%	60.8% (95% c.i. 54.9–66.4%)	Potentially feasible with modifications (yellow)
LED adherence: number of LED doses taken divided by the number of doses prescribed for each patient in the intervention arm	≥80%	81.7% (95% c.i. 74.1%,89.3%)	Feasible (green)
Follow-up completion: completion of the pre-LED, post-LED, and 30-day postoperative visits, along with complete anthropometric measurements and study questionnaires	≥90%	89.0% (95% c.i. 80.7%,94.6%)	Potentially feasible with modifications (yellow)
Network development: number of sites recruited from/partnered with throughout the duration of the trial	10	6	Not feasible (red)

LED, low-energy diet.

A total of 288 patients who were eligible were approached and, in total, 175 of them consented to be randomized. Only 91 were ultimately randomized and the remaining 74 were still awaiting a surgery date at the time that the trial closed. Altogether, 60.8% (60.8% (95% c.i. 54.9-66.4%)) of the eligible patients approached, consented and agreed to be randomized (175 of 288). This fell within the yellow light criteria for feasibility, thus potentially supporting feasibility of a definitive trial if modifications are made.

Of the 91 patients randomized, 3 patients withdrew from the study after randomization (due to not wanting to participate in the control arm) and 7 patients had at least one outcome data point missing. Thus, 81 of the original 91 patients had complete follow-up data, equating to a follow-up completion of 89.0% (95% c.i. 80.7% to 94.6%). This also potentially supports the feasibility of a definitive trial with modifications.

Adherence to the LED protocol was 81.7% (95% c.i. 74.1% to 89.3%), falling within the green light criteria and supporting feasibility of a definitive trial. Adherence gradually decreased throughout the duration of the LED protocol. Detailed adherence data are reported in *Table 4*.

**Table 4:** Adherence and LED-associated adverse event data

Outcome	LED group (*n* = 45)
Full LED prescribed	40 (88.9)
LED duration (days), mean(s.d.)	20.9(0.4)
**Adherence**	
Percentage of doses taken, mean(s.d.)*	81.7(25.3)
First week, mean(s.d.)*	86.4(18.4)
Second week, mean(s.d.)*	82.6(25.7)
Third week, mean(s.d.)*	79.4(28.5)
All prescribed doses taken	11 (24.4)
Did not eat above protocol	7 (15.6)
Ate above protocol	4 (8.9)
Food eaten above protocol	29 (64.4)
Number of days food eaten above protocol, median (i.q.r.)	2 (0–6)
Reasons for non-adherence†, *n* of *n* (%)	
Hunger	8 of 33 (24.2)
Taste	2 of 33 (6.1)
Adverse event(s)	5 of 33 (15.2)
Not seeing change	1 of 33 (3.0)
Did not want to	2 of 33 (6.1)
Too full	4 of 33 (12.1)
Other/circumstantial	11 of 33 (33.3)
**Adverse events**	
All adverse events	31 (68.9)
Minor	31 (68.9)
Constipation	11 (24.4)
Diarrhoea	4 (9.1)
Nausea	6 (13.3)
Fatigue	9 (20.0)
Dizziness	5 (11.4)
Headache	7 (15.6)
Alopecia	1 (2.2)
Major	0 (0.0)

Values are *n* (%) unless otherwise indicated. *Corrected for number of doses prescribed. †A total of 33 of 38 patients who did not completely adhere to the LED protocol reported reasons for non-adherence. LED, low-energy diet; i.q.r., interquartile range.

Lastly, at study completion, the network for this trial included six sites: the four sites that were recruited from as well as two other international sites completing similar research programmes, interested in collaborating further for a definitive trial. This fell below the criteria for feasibility (that is 10 sites).

### Safety outcomes

Minor adverse events were experienced by 31 of the 45 patients randomized to the LED group (68.9%) (*Table 4*). The most common minor adverse events were constipation (11 patients (24.4%)), fatigue (9 patients (20.0%)), and headache (7 patients (15.6%)). None of the patients experienced major LED-associated adverse events. No adverse events were reported in the control group.

### Clinical outcomes

Intervention-interval anthropometric outcome data are reported in *Table 5*. Patients in the LED group lost 4.6 (95% c.i. 3.5 to 5.6) kg more and had their waist circumference reduced by 3.6 (95% c.i. 1.5 to 5.7) cm more during the 3-week intervention interval than patients in the control group. Grip strength changes during the 3-week intervention interval were similar for both groups.

**Table 5:** Intervention-interval anthropometric outcome data

Outcome	LED group (baseline *n* = 45, post-LED *n* = 45, and postoperative *n* = 44)	Control group (baseline *n* = 46, post-LED *n* = 43, and postoperative *n* = 42)	MD (95% c.i.)*
**Weight (kg)**			
Baseline	107.8(21.5)	105.7(18.4)	−4.6 (−5.6,−3.5)
Post-LED	102.0(20.8)	104.9(18.6)	
**BMI (kg/m^2^)**			
Baseline	36.1(5.6)	36.4(6.4)	−1.8 (−2.3,−1.3)
Post-LED	33.9(5.1)	36.1(6.7)	
**Waist circumference (cm)**			
Baseline	118.5(13.4)	116.3(12.6)	−3.6 (−5.7,−1.5)
Post-LED	114.6(13.2)	116.6(14.8)	
**Grip strength (kg)**			
Baseline	30.3(12.0)	25.1(9.1)	−0.1 (−1.8,+1.6)
Post-LED	29.8(11.4)	25.8(8.6)	

Values are mean(s.d.) unless otherwise indicated. *LED group as reference group in analysis of covariance (ANCOVA). LED, low-energy diet; MD, mean difference.

Postoperative-interval anthropometric outcome data are reported in *Table S3*. Greater weight loss and waist circumference decreases were maintained in the LED group compared with the control group throughout the entirety of the trial follow-up interval.

Quality-of-life and clinical outcome data are reported in *Table 6*. Mean SF-36 general health scores increased in both groups during the intervention interval as well as from baseline to the end of trial follow-up. One less patient in the LED group experienced a postoperative complication. One patient in the LED group died in the 30-day postoperative interval after undergoing a small bowel resection and hepatectomy for a metastatic neuroendocrine tumour and experiencing an anastomotic leak. There was no evidence of differences in postoperative LOS and operating time between groups. Patients in the LED group lost less blood intraoperatively.

**Table 6:** Quality-of-life and clinical outcome data

Outcome	LED group	Control group	MD/RD (95% c.i.)*
Quality of life	Baseline *n* = 45, post-LED *n* = 45, and postoperative *n* = 44	Baseline *n* = 46, post-LED *n* = 43, and postoperative *n* = 42	
SF-36 general health, mean(s.d.)			
Intervention-interval change			+0.4 (−4.8,+5.6)
Baseline	62.9(18.5)	56.1(20.8)	
Post-LED	66.3(18.8)	60.5(20.9)	
Postoperative-interval change			+2.7 (−2.7,+8.2)
Baseline	62.9(18.5)	56.1(20.8)	
After surgery	67.4(16.2)	59.4(21.1)	
**Clinical**	*n* = 44	*n* = 41	
Overall 30-day postoperative morbidity	13 (29.5)	14 (33.3)	−3.8% (−23.6%,+16.0%)
Thirty-day postoperative mortality	1 (2.3)	0 (0.0)	+2.3% (−2.1%,+6.7%)
Postoperative LOS (days), mean(s.d.)	1.7(3.2)	2.0(2.5)	−0.3 (−1.5,1.0)
Operating time (min), mean(s.d.)	124.7(123.4)	130.0(101.4)	−5.3 (−54.2,43.7)
Estimated intraoperative blood loss (ml), mean(s.d.)	269.6(298.5)	320.9(459.7)	−51.3 (−271.6,169.0)
Intraoperative complication	4 (9.1)	4 (9.5)	−0.4% (−12.8%,12.1%)

Values are *n* (%) unless otherwise indicated. *LED group as reference group in analysis of covariance (ANCOVA). LED, low-energy diet; MD, mean difference; RD, risk difference; SF-36, Short-Form 36; LOS, length of stay.

### Meta-analysis

**Fig. 2:**
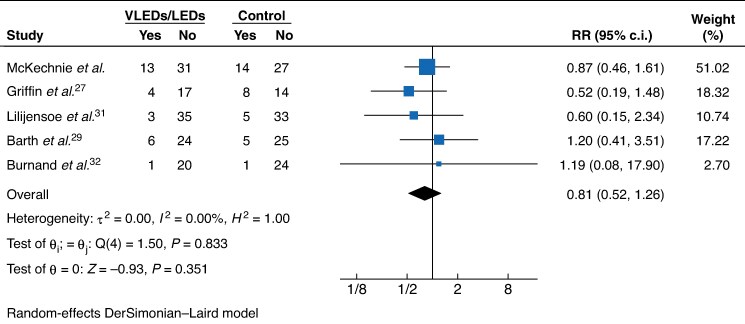
Forest plot for a random-effects meta-analysis comparing preoperative VLEDs/LEDs with control in terms of 30-day postoperative morbidity VLEDs, very low-energy diets; LEDs, low-energy diets; RR, risk ratio.

The meta-analysis included four RCTs from the authors’ previous systematic review as well as an additional pilot RCT^27^ identified in the updated search. Five RCTs, including the present trial, reported comparative data for 30-day postoperative morbidity. Study details are reported in *Table S4*. In a pooled analysis, patients in the intervention group had an 19% relative risk reduction in 30-day postoperative morbidity compared with the control group (RR 0.81 (95% c.i. 0.52 to 1.26); *P* = 0.351; *I*^2^ = 0%) (*Fig. 2*).

## Discussion

Patients living with a BMI >30 kg/m^2^ undergoing elective non-MBS intra-abdominal surgery or orthopaedic surgery can experience significant preoperative weight loss with just a 3-week course of preoperative LEDs. The prescription of LEDs is safe, is well tolerated, has acceptable adherence, and does not decrease lean body mass in this patient population. The predefined feasibility targets for recruitment rate, randomization percentage, and complete follow-up were not met. A hypothesis-generating pooled analysis for 30-day postoperative morbidity suggested a potentially clinically relevant benefit; however, a wide 95% confidence interval and the resulting risk of Type II error introduced uncertainty. As such, additional sites to address concerns regarding recruitment in a definitive trial aimed at evaluating the clinical efficacy of preoperative VLEDs/LEDs in this setting should be considered.

Despite the present study being designed as a pilot trial, and thus perhaps not generalizing perfectly to previously published data, it was the largest randomized evaluation of preoperative LEDs for patients undergoing elective non-MBS to date. The LED protocol was pragmatic and can likely be easily and safely implemented into clinical practice in a wide variety of settings. Moreover, the best practices for conducting and reporting pilot randomized trials were adhered to^20,25^. In addition to these strengths, the present study also has several limitations. First, approximately 11% of the sample did not have complete follow-up data. These missing data could potentially bias a definitive trial through attrition bias. To prevent attrition and post-randomization dropouts in the future, studies evaluating LEDs and similar interventions should consider including a more standardized control arm that offers some additional benefit to patients (for example, nutritional counselling with a dietitian, exercise regimen, etc.) to prevent resentful demoralization and resulting dropout, or consider cluster randomization^28^. The study sample was heterogeneous, as it included both patients undergoing intra-abdominal surgery for a variety of indications as well as patients undergoing lower-extremity orthopaedic surgery. Thus, the resulting clinical outcomes are difficult to interpret given the different baseline risks between these two patient populations and should be regarded as exploratory in nature. However, by including both patients undergoing intra-abdominal surgery and patients undergoing orthopaedic surgery, it was possible to increase the sample size and evaluate the feasibility of this intervention across different patient populations that may benefit from this intervention. If a definitive trial were to include both of these patient populations, it would have to be powered for subgroup analyses based on type of surgery. Despite being the largest trial to date, the sample size remained relatively small. Therefore, it was not possible to effectively compare the intervention group with the control group in terms of clinical efficacy outcomes, nor was it possible to achieve prognostic balance across the two groups (for example, significant imbalances in types of operations and indications for operations). However, the sample size was effective for evaluating feasibility outcomes, the primary focus. Lastly, the control group was unblinded given the nature of the intervention, thus raising concerns with regard to performance bias. Mostly objective measures were investigated as part of this trial to counteract this limitation.

There have been five other RCTs evaluating preoperative VLEDs/LEDs as a means of optimizing patients living with a BMI >30 kg/m^2^ before non-MBS^27,29–32^. The most recent was a pilot RCT similar to the present study conducted by Griffin *et al*.^27^ in Australia. Griffin *et al*.^27^ included 51 patients undergoing elective laparoscopic cholecystectomy, hernia repair, or total abdominal hysterectomy with a BMI >30 kg/m^2^. Patients undergoing laparoscopic cholecystectomy received a 2-week dietician-led VLED/LED programme and patients undergoing hernia repair or total abdominal hysterectomy received a 3–12-week dietician-led VLED/LED programme (800–920 kcal/day). Griffin *et al*.^27^ focused on feasibility outcomes. Similar to the present trial, the randomization percentage (20%) and attrition (21%) fell below their feasibility thresholds. Griffin *et al*.^27^ were also able to demonstrate good adherence to their intervention (88%), which was very similar to the present study. None of the 23 patients randomized to the intervention experienced a major adverse event and lean body mass was maintained. Weight loss was 4.6 kg greater and waist circumference reduction was 7.4 cm greater in the intervention group. These data, in conjunction with the data from the present study, support the notion that preoperative VLEDs/LEDs in the setting of elective non-MBS are safe and effective. Liljensøe *et al*.^31^ performed the largest RCT before the present study, evaluating 76 patients with a BMI >30 kg/m^2^ scheduled for total knee arthroplasty for knee osteoarthritis. The 38 patients randomized to the intervention group received an 8-week VLED programme consisting of a commercially available liquid formula (Cambridge Weight Plan^®^, Cambridge, UK) providing just over 800 kcal/day. Patients in the intervention group lost 10.7 kg, the majority of which was fat mass (6.7 kg). The VLED group also demonstrated biochemical-profile improvements compared with the control group, with significant reductions in total cholesterol, low-density lipoprotein (LDL), and triglycerides. Adjusted analyses demonstrated similar patient-reported quality-of-life SF-36 scores across groups and the risk of postoperative complication was low (intervention group: 2 of 38; control group: 3 of 38). The present trial also found a relatively low proportion of orthopaedic patients living with excess weight experiencing postoperative complications (intervention group: 4 of 12; control group: 1 of 5). Interestingly, patients in the Liljensøe *et al*.^31^ trial were followed for a total of 12 months after surgery and patients in the LED group maintained their weight loss during this time frame.

In terms of postoperative clinical outcomes, previous trials have been slightly more heterogeneous. In the present meta-analysis of 30-day overall postoperative morbidity, quantitative heterogeneity was not identified (*I*^2^ = 0%), yet there was some qualitative heterogeneity. Two of the earlier RCTs, by Burnand *et al*.^32^ and Barth *et al*.^29^, evaluating patients undergoing laparoscopic cholecystectomy and hepatectomy respectively, had point estimates that favoured the control group, with wide 95% confidence intervals, while the three most recent trials, including the two largest trials evaluating preoperative VLEDs/LEDs for patients undergoing non-MBS to date, had point estimates that favoured the intervention group. Nonetheless, the present study’s pooled estimate (RR 0.81 (95% c.i. 0.52 to 1.26)) was similar to previous pooled estimates for the bariatric surgery population (RR 0.67 (95% c.i. 0.39 to 1.17)), for whom it is purported that VLEDs may reduce postoperative complications^15^. Altogether, uncertainty remains as to whether there is a clinically significant benefit regarding the use of preoperative VLEDs/LEDs in terms of this important patient outcome, but the present study’s point estimate suggests that a properly powered study may uncover potential benefits.

One of the concerns regarding the use of a dieting programme such as preoperative LEDs is that they are short-term lifestyle interventions that may not allow for sustained weight loss and healthy habits. While they may induce significant preoperative weight loss, the lack of sustained use of these programmes may subsequently result in weight regain shortly after surgery. The data from the present study suggest that weight loss is maintained at least in the acute postoperative interval, with weight at the post-intervention visit (LED group: mean(s.d.) of 102.0(20.8) kg; control group: mean(s.d.) of 104.9(18.6) kg) being nearly identical to weight at the 30-day postoperative visit (LED group: mean(s.d.) of 102.0(20.6) kg; control group: mean(s.d.) of 104.6(18.8) kg). However, some of this sustained weight loss could be due to the physiological stress associated with surgery, and the loss of lean body mass during the acute postoperative interval^33,34^. Long-term data from Liljensøe *et al*.^31^ suggest that weight loss can be maintained for up to 1 year after surgery. LEDs used as part of a medical weight loss programme for patients living with a BMI >30 kg/m^2^ can maintain total body weight loss percentages upwards of 15% at 18 months^35^. However, these medical weight loss programmes include gradual, stepwise reintroduction of a healthy solid food diet under the supervision of a registered dietitian. While programmes involving a registered dietitian likely afford more expertise, data from the DiRECT and DROPLET trials suggest that nurses and lay counsellors respectively can effectively lead programmes with comparable amounts of long-term weight loss to dietitian-led programmes^36,37^. Regardless, the degree to which weight loss is sustained long-term with these interventions remains uncertain. A systematic review of nine RCTs evaluating VLEDs for weight loss reported significant heterogeneity in 1-year weight regain, ranging from −7% to 122%, highlighting the uncertainty pertaining to the long-term implications of these programmes^38^. A more recent systematic review including 23 RCTs suggested that LEDs consistently resulted in larger amounts of weight loss compared with different types of food-based diets at 1-year follow-up^39^. Further study of multidisciplinary nutritional optimization programmes is required to identify the best approach for both short-term preoperative weight loss and sustained long-term postoperative weight loss.

Contemporary multidisciplinary nutritional optimization programmes for patients living with obesity must at the very least also consider OMMs. Since the STEP 1 publication in 2021, the use of glucagon-like peptide-1 (GLP-1) agonists, such as semaglutide and liraglutide, has become increasingly ubiquitous^40^. The quality of data supporting their use specifically in the preoperative setting is of lower certainty compared with data supporting their use for medical weight loss. A recent meta-analysis found 21 studies evaluating the use of GLP-1 before elective surgery and the pooled analysis suggested that the risk of postoperative complications may be lower for patients using these medications (OR 0.78 (95% c.i. 0.59 to 1.05))^41^. While there were originally concerns regarding an increased risk of aspiration at the time of anaesthetic induction due to delayed gastric emptying as a result of these medications, 2024 multisociety anaesthesia clinical practice guidelines recommend that GLP-1 agonists can be continued before surgery for patients not at risk of delayed gastric emptying^42^. Though slightly ambiguous, these recommendations will likely allow for continued OMM use before elective surgery more frequently, thus highlighting the need for more robust data evaluating their use in this setting moving forward.

Overall, these data from the largest RCT evaluating preoperative LEDs for patients living with a BMI >30 kg/m^2^ undergoing elective non-MBS intra-abdominal or orthopaedic surgery suggest that this intervention can induce significant preoperative weight loss. While LEDs were safe, were well tolerated, and had acceptable adherence, the recruitment and retention data do not support the conduct of a definitive trial in its present form. Recruitment rate, randomization percentage, and follow-up completion would need to be enhanced to allow for the feasible completion of a definitive trial. Adding additional sites, increasing the robustness of the standard-care arm, and altering the follow-up schedule are options for modifying the methodology that might enhance feasibility and allow for conduct of a definitive trial. Moreover, a definitive trial should likely focus on a single patient population to address concerns regarding heterogeneity identified in the present study. Pooled analyses of clinical efficacy data from the current evidence base of RCTs, while exploratory in nature, suggest there may be a benefit in terms of postoperative morbidity. Thus, a definitive trial should be considered.

## Supplementary Material

znag023_Supplementary_Data

## Data Availability

The data that support the findings of this study are available on request from the corresponding author. The data are not publicly available due to restrictions (for example containing information that could compromise the privacy of research participants).
