# Supplementary material for: Preoperative low-energy diets for patients with a body mass index >30 kg/m2 undergoing non-bariatric surgery: pilot feasibility randomized clinical trial and a systematic review and meta-analysis of efficacy data
Source: Br J Surg. 2026 Mar 13;113(5):znag023. doi: 10.1093/bjs/znag023 (PMC13155937; doi:10.1093/bjs/znag023)
Supplement: znag023_Supplementary_Data [file znag023_supplementary_data.zip › PREPARE_MainManuscript_Appendix5.docx]

Database:
Embase <1974 to 2021 June 13> 
OVID Medline Epub Ahead of Print, In-Process & Other Non-Indexed Citations, Ovid MEDLINE(R) Daily and Ovid MEDLINE(R) 1946 to Present

| # | Query |
| --- | --- |
| 1 | Non-Bariatric surgery.mp. |
| 2 | Nonbariatric surgery.mp. |
| 3 | preoperative diet*.mp. |
| 4 | pre-operative diet*.mp. |
| 5 | preoperative weight loss.mp. |
| 6 | pre-operative weight loss.mp. |
| 7 | exp Colorectal Surgery/ae, di, mt, mo, st, su [Adverse Effects, Diagnosis, Methods, Mortality, Standards, Surgery] |
| 8 | hepatobiliary surgery.mp. |
| 9 | Oncologic surgery.mp. |
| 10 | exp Thoracic Surgery/ae, co, mt, mo, st [Adverse Effects, Complications, Methods, Mortality, Standards] |
| 11 | exp Gynecologic Surgical Procedures/ae, mt, mo, st [Adverse Effects, Methods, Mortality, Standards] |
| 12 | exp Urologic Surgical Procedures/ae, mt, mo, st [Adverse Effects, Methods, Mortality, Standards] |
| 13 | Liver Transplantation/ or Kidney Transplantation/ or Transplant surgery.mp. or Heart Transplantation/ |
| 14 | Mastectomy/ or Breast surgery.mp. or Mastectomy, Segmental/ or Mammaplasty/ |
| 15 | Foregut surgery.mp. |
| 16 | Digestive System Surgical Procedures/ae, mt, mo, st [Adverse Effects, Methods, Mortality, Standards] |
| 17 | Laparoscopic surgery.mp. |
| 18 | exp Minimally Invasive Surgical Procedures/ae, mt, mo, st [Adverse Effects, Methods, Mortality, Standards] |
| 19 | exp Laparoscopy/ae, co, mt, mo, ph, st, su, tr [Adverse Effects, Complications, Methods, Mortality, Physiology, Standards, Surgery, Transplantation] |
| 20 | exp Robotic Surgical Procedures/ae, mt, st [Adverse Effects, Methods, Standards] |
| 21 | exp Orthopedic Procedures/ae, mt, mo, rh, st [Adverse Effects, Methods, Mortality, Rehabilitation, Standards] |
| 22 | exp Surgery, Plastic/ae, co, mt, mo, rh, st [Adverse Effects, Complications, Methods, Mortality, Rehabilitation, Standards] |
| 23 | exp Otorhinolaryngologic Surgical Procedures/ae, mt, mo, st [Adverse Effects, Methods, Mortality, Standards] |
| 24 | exp Vascular Surgical Procedures/ae, mt, mo, st [Adverse Effects, Methods, Mortality, Standards] |
| 25 | exp Natural Orifice Endoscopic Surgery/ae, mt, mo, st [Adverse Effects, Methods, Mortality, Standards] |
| 26 | *Preoperative Care/ae, ed, me, mt, mo, st [Adverse Effects, Education, Metabolism, Methods, Mortality, Standards] |
| 27 | *Postoperative Complications/bl, ci, co, di, dh, ep, me, mo, ph, pp, su [Blood, Chemically Induced, Complications, Diagnosis, Diet Therapy, Epidemiology, Metabolism, Mortality, Physiology, Physiopathology, Surgery] |
| 28 | Optifast.mp. |
| 29 | Very-low energy diet*.mp. |
| 30 | Low energy diet*.mp. |
| 31 | Very-low calorie diet*.mp. |
| 32 | Low calorie diet*.mp. |
| 33 | Weight loss program*.mp. |
| 34 | Weight loss intervention*.mp. |
| 35 | Weight loss therapy.mp. |
| 36 | Dietary restriction.mp. |
| 37 | Caloric restriction.mp. or *Caloric Restriction/ |
| 38 | Food, Formulated/ or Formula diet.mp. |
| 39 | Total diet replacement.mp. |
| 40 | Liquid low-calorie diet.mp. |
| 41 | or/1-27 |
| 42 | or/28-40 |
| 43 | 41 and 42 |

**Appendix 3.** Complete Search Strategy.
