# Supplementary material for: Preoperative low-energy diets for patients with a body mass index >30 kg/m2 undergoing non-bariatric surgery: pilot feasibility randomized clinical trial and a systematic review and meta-analysis of efficacy data
Source: Br J Surg. 2026 Mar 13;113(5):znag023. doi: 10.1093/bjs/znag023 (PMC13155937; doi:10.1093/bjs/znag023)
Supplement: znag023_Supplementary_Data [file znag023_supplementary_data.zip › PREPARE_Manuscript_Appendix3.docx]

**
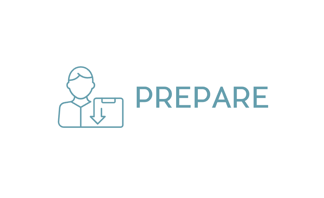
**

**PReoperative low-Energy diets for PAtients living with excess weight undergoing non-bariatric surgery Randomized Evaluation (PREPARE): A Pilot Randomized Controlled Trial**

*Very Low Energy Diet Instructions*

**Low Energy Diet Instructions**

**PREPARE Trial**

**How do I take the weight loss supplement?**

1. Be sure to consume 4 Optifast® 900 or Medimeal® packets each day.
2. Take 1 sachet of your Optifast® 900 or Medimeal® once in the morning (AM), once at noon, once in the early evening (PM #1), and once later in the evening (PM #2) for three-weeks starting 23 days prior to your surgery date.
3. Mix 1 sachet with at least 300 mL (1 ¼ cups) of cold water. You may add ice cubes if you’d like. Shake well or blend in a blender.

**What else can I take with my weight loss supplement?**

1. Drink at least 2 L (8 cups) of any combination of the following each day in addition to the Optifast® 900 or Medimeal®:
   1. Water
   2. Coffee or tea (minimal milk or sugar added)
      1. Artificial sweetener may be added
   3. Crystal light or Mio©
   4. Low fat broth (up to 20 kcal per serving is allowed)
   5. Sugar-free Jello
2. Can consume up to a total of 500 mL (2 cups) a day of the following while taking Optifast® 900 or Medimeal®:
   1. Green peppers
   2. Broccoli
   3. Cauliflower
   4. Lettuce
   5. Spinach
   6. Celery
   7. Cabbage
   8. Cucumber
      1. *Can add up to 15 mL (1 tablespoon) of calorie-free dressing
3. You cannot consume any other solid food while consuming Optifast® 900 or Medimeal®
