# Supplementary material for: Preoperative low-energy diets for patients with a body mass index >30 kg/m2 undergoing non-bariatric surgery: pilot feasibility randomized clinical trial and a systematic review and meta-analysis of efficacy data
Source: Br J Surg. 2026 Mar 13;113(5):znag023. doi: 10.1093/bjs/znag023 (PMC13155937; doi:10.1093/bjs/znag023)
Supplement: znag023_Supplementary_Data [file znag023_supplementary_data.zip › PREPARE_Manuscript_Main_Appendix4.docx]

| **Activity** | **Before Randomization** | **-23** | **After Randomization** | | | |
| --- | --- | --- | --- | --- | --- | --- |
|  | **-60 to -23** |  | **-23 to -2** | **0** | **0 to discharge** | **+30** |
| Eligibility assessment | X |  |  |  |  |  |
| Informed consent | X |  |  |  |  |  |
| Randomization |  | X |  |  |  |  |
| Intervention (i.e., LED) |  |  | X |  |  |  |
| Baseline demographic data | X |  |  |  |  |  |
| Operative data collection |  |  |  | X |  |  |
| Baseline anthropometrics | X |  |  |  |  |  |
| Post-intervention anthropometrics |  |  |  | X |  |  |
| Post-surgical anthropometrics |  |  |  |  |  | X |
| Intervention-associated adverse events |  |  | X | X | X | X |
| Postoperative morbidity |  |  |  |  | X | X |
| QoL assessment (i.e., SF-36) | X |  |  | X |  | X |
| Intervention Adherence |  |  | X | X |  |  |
| Follow-up completion |  |  |  |  |  | X |

*LED, low energy diet; QoL, quality of life*

*Appendix 2.* Standard Protocol Items: Recommendations for Interventional Trials (SPIRIT) figure for participant timeline
