# Supplementary material for: Preoperative low-energy diets for patients with a body mass index >30 kg/m2 undergoing non-bariatric surgery: pilot feasibility randomized clinical trial and a systematic review and meta-analysis of efficacy data
Source: Br J Surg. 2026 Mar 13;113(5):znag023. doi: 10.1093/bjs/znag023 (PMC13155937; doi:10.1093/bjs/znag023)
Supplement: znag023_Supplementary_Data [file znag023_supplementary_data.zip › PREPARE_Tables.docx]

**Table 1.** Patient characteristics

| **Characteristic** | **LED**  **n=45** | **Control**  **n=46** |
| --- | --- | --- |
| Age, years (mean [SD]) | 58.6 (11.3) | 55.1 (12.9) |
| Female (n [%]) | 21 (46.7) | 30 (65.2) |
| BMI, kg/m^2^ (mean [SD]) | 36.1 (5.6) | 36.4 (6.4) |
| Weight, kg (mean [SD]) | 107.8 (21.5) | 105.7 (18.4) |
| Waist circumference, cm (mean [SD]) | 118.5 (13.4) | 116.3 (12.6) |
| Grip strength, kg (mean [SD]) | 30.3 (12.0) | 25.1 (9.1) |
| Race (n [%]) |  |  |
| White | 40 (88.9) | 43 (93.5) |
| Black | 2 (4.4) | 1 (2.2) |
| Latin American | 2 (4.4) | 1 (2.2) |
| Arab | 1 (2.2) | 1 (2.2) |
| Ethnicity (n [%]) |  |  |
| North American Origin | 36 (80.0) | 32 (69.6) |
| European Origin | 7 (15.6) | 12 (26.1) |
| Latin, Central, or South American Origin | 2 (4.4) | 1 (2.2) |
| Asian Origin | 0 (0.0) | 1 (2.2) |
| Smoker (n [%]) | 4 (8.9) | 9 (19.6) |
| CCI (median [IQR]) | 2 (1-3) | 2 (0-4) |
| mFI-11 (median [IQR]) | 0 (0-1) | 1 (0-1) |
| ASA class (median [IQR]) | 3 (3-3) | 3 (3-4) |
| Diabetes (n [%]) | 7 (15.6) | 8 (17.4) |
| History of previous surgery in same surgical field (n [%]) | 18 (40.0) | 23 (50.0) |

*n, Number of Patients; LED, Low Energy Diet; SD, Standard Deviation; IQR, Interquartile Range; BMI, Body Mass Index; kg, Kilograms; m, Meters; cm, Centimeters; CCI, Charlson Comorbidity Index; mFI-11, Modified Frailty Index; ASA, American Society of Anesthesiologists*

**Table 2.** Disease and treatment characteristics

| **Characteristic** | **LED**  **n=45** | **Control**  **n=46** |
| --- | --- | --- |
| *Disease Characteristics* |  |  |
| Disease (n [%]) |  |  |
| Colorectal neoplasia | 5 (11.1) | 11 (23.9) |
| Diverticular disease | 1 (2.2) | 2 (4.3) |
| Stoma status | 2 (4.4) | 1 (2.2) |
| Rectal prolapse | 1 (2.2) | 0 (0.0) |
| Benign biliary disease | 11 (24.4) | 14 (30.4) |
| Abdominal wall hernia | 10 (22.2) | 8 (17.4) |
| Hiatal hernia | 0 (0.0) | 1 (2.2) |
| GIST | 1 (2.2) | 2 (4.3) |
| NET | 1 (2.2) | 0 (0.0) |
| Endometrial cancer | 1 (2.2) | 0 (0.0) |
| Osteoarthritis | 12 (26.7) | 7 (15.2) |
| Malignant disease (n [%]) | 7 (15.6) | 13 (28.3) |
| *Treatment Characteristics* |  |  |
| Procedure (n [%]) |  |  |
| Colectomy | 5 (11.1) | 5 (10.9) |
| Proctectomy | 2 (4.4) | 5 (10.9) |
| Ileostomy reversal | 0 (0.0) | 1 (2.2) |
| Colostomy reversal | 2 (4.4) | 0 (0.0) |
| Gastrectomy | 1 (2.2) | 1 (2.2) |
| Pancreaticoduodenectomy | 0 (0.0) | 1 (2.2) |
| Hepatectomy | 1 (2.2) | 3 (6.5) |
| Cholecystectomy | 11 (24.4) | 14 (30.4) |
| Hysterectomy | 1 (2.2) | 0 (0.0) |
| Hernia repair with mesh | 8 (17.8) | 5 (10.9) |
| Hernia repair without mesh | 2 (4.4) | 3 (6.5) |
| Hiatal hernia repair | 0 (0.0) | 1 (2.2) |
| THA | 3 (6.7) | 3 (6.5) |
| TKA | 9 (20.0) | 4 (8.7) |
| MIS (n [%])* | 24/33 (72.7) | 30/39 (76.9) |
| MIS converted to open (n [%])* | 1/33 (4.2) | 0/39 (0.0) |
| Spinal anesthetic (n [%]) | 13 (29.5) | 4 (9.8) |

*intra-abdominal surgery only

*n, Number of Patients; LED, Low Energy Diet; GIST, Gastrointestinal Stromal Tumor; NET, Neuroendocrine Tumor; THA, Total Hip Arthroplasty; TKA, Total Knee Arthroplasty; MIS, Minimally Invasive Surgery*

**Table 3.** Feasibility outcomes and stoplight criteria.

| **Feasibility Criteria** | **Definition of Success** | **Feasibility Data** | **Interpretation** |
| --- | --- | --- | --- |
| *Recruitment rate:* number of patients randomized per month | 16 patients per month | 9.4 patients recruited per month | Not feasible  (Red) |
| *Randomization percentage:* number of patients agreeing to be randomized divided by the number of eligible patients approached | ≥70% | 57.3% (95%CI 51.4-63.1%) | Potentially feasible with modifications  (Yellow) |
| *LED adherence:* number of LED doses taken divided by the number of doses prescribed for each patient in the intervention arm | ≥80% | 81.7% (95%CI 74.1-89.3%) | Feasible  (Green) |
| *Follow-up completion:* completion of the pre-LED, post-LED, and 30-day postoperative visits, along with complete anthropometric measures and study questionnaires | ≥90% | 89.0% (95%CI 80.7-94.6%) | Potentially feasible with modifications  (Yellow) |
| *Network development:* number of sites recruited from/partnered with throughout the duration of the trial | 10 | 6 | Not feasible  (Red) |

*LED, Low Energy Diet; CI, Confidence Interval*

**Table 4.** Adherence and LED associated adverse event data

| **Outcome** | **LED**  **n=45** |
| --- | --- |
| Full LED prescribed (n [%]) | 40 (88.9) |
| LED duration, days (mean [SD]) | 20.9 (0.4) |
| *Adherence* |  |
| Percentage of Doses Taken (mean [SD])* | 81.7 (25.3) |
| 1^st^ week (mean [SD])* | 86.4 (18.4) |
| 2^nd^ week (mean [SD])* | 82.6 (25.7) |
| 3^rd^ week (mean [SD])* | 79.4 (28.5) |
| All prescribed doses taken (n [%]) | 11 (24.4) |
| Did not eat above protocol | 7 (15.6) |
| Ate above protocol | 4 (8.9) |
| Food eaten above protocol (n [%]) | 29 (64.4) |
| Number of days food eaten above protocol (median [IQR]) | 2 (0-6) |
| Reasons for non-adherence (n [%])** |  |
| Hunger | 8/33 (24.2) |
| Taste | 2/33 (6.1) |
| Adverse event(s) | 5/33 (15.2) |
| Not seeing change | 1/33 (3.0) |
| Did not want to | 2/33 (6.1) |
| Too full | 4/33 (12.1) |
| Other/circumstantial | 11/33 (33.3) |
| *Adverse Events* |  |
| All adverse events | 31 (68.9) |
| Minor (n [%]) | 31 (68.9) |
| Constipation | 11 (24.4) |
| Diarrhea | 4 (9.1) |
| Nausea | 6 (13.3) |
| Fatigue | 9 (20.0) |
| Dizziness | 5 (11.4) |
| Headache | 7 (15.6) |
| Alopecia | 1 (2.2) |
| Major (n [%]) | 0 |

*corrected for number of doses prescribed

**33 of 38 patients who did not completely adhere to LED protocol reported reasons for non-adherence

*n, Number of Patients; SD, Standard Deviation; IQR, Interquartile Range; LED, Low Energy Diet*

**Table 5.** Intervention period anthropometric outcome data

| **Outcome** | **LED**  *Baseline n=45*  *Post-LED n=45*  *Postoperative n=44* | **Control**  *Baseline n=46*  *Post-LED n=43*  *Postoperative n=42* | **MD (95%CI)*** |
| --- | --- | --- | --- |
| Weight, kg (mean [SD]) |  |  |  |
| Baseline | 107.8 (21.5) | 105.7 (18.4) | -4.5 (-5.6 to -3.5) |
| Post-LED | 102.0 (20.8) | 104.9 (18.6) |  |
| BMI, kg/m^2^ (mean [SD]) |  |  |  |
| Baseline | 36.1 (5.6) | 36.4 (6.4) | -1.8 (-2.3 to -1.3) |
| Post-LED | 33.9 (5.1) | 36.1 (6.7) |  |
| Waist circumference, cm (mean [SD]) |  |  |  |
| Baseline | 118.5 (13.4) | 116.3 (12.6) | -3.6 (-5.7 to -1.5) |
| Post-LED | 114.6 (13.2) | 116.6 (14.8) |  |
| Grip strength, kg (mean [SD]) |  |  |  |
| Baseline | 30.3 (12.0) | 25.1 (9.1) | -0.1 (-1.8 to 1.6) |
| Post-LED | 29.8 (11.4) | 25.8 (8.6) |  |

*LED group as reference group in analysis of covariance (ANCOVA)

*n, Number of Patients; LED, Low Energy Diet; SD, Standard Deviation; kg, Kilograms; m, Meters; cm, Centimeters; MD, Mean Difference, CI, Confidence Interval*s

**Table 6.** Quality of life and clinical outcome data

| **Outcome** | **LED** | **Control** | **MD/RD (95% CI)*** |
| --- | --- | --- | --- |
| *Quality of Life* | *Baseline n=45*  *Post-LED n=45*  *Postoperative n=44* | *Baseline n=46*  *Post-LED n=43*  *Postoperative n=42* |  |
| SF-36 General Health (mean [SD]) |  |  |  |
| Intervention Period Change |  |  | +0.4 (-4.8 to 5.6) |
| Baseline | 62.9 (18.5) | 56.1 (20.8) |  |
| Post-LED | 66.3 (18.8) | 60.5 (20.9) |  |
| Postoperative Period Change |  |  | +2.7 (-2.7 to 8.2) |
| Baseline | 62.9 (18.5) | 56.1 (20.8) |  |
| Postoperatively | 67.4 (16.2) | 59.4 (21.1) |  |
| *Clinical* | *n=44* | *n=41* |  |
| Overall 30-day postoperative morbidity (n [%]) | 13 (29.5) | 14 (33.3) | -3.8% (-23.6 to 16.0) |
| 30-day postoperative mortality (n [%]) | 1 (2.3) | 0 (0.0) | +2.3% (-2.1 to 6.7) |
| Postoperative LOS, days (mean [SD]) | 1.7 (3.2) | 2.0 (2.5) | -0.3 (-1.5 to 1.0) |
| Operative time, minutes (mean [SD]) | 124.7 (123.4) | 130.0 (101.4) | -5.3 (-54.2 to 43.7) |
| Estimated intraoperative blood loss, mL (mean [SD]) | 269.6 (298.5) | 320.9 (459.7) | -51.3 (-271.6 to 169.0) |
| Intraoperative complication (n [%]) | 4 (9.1) | 4 (9.5) | -0.4% (-12.8 to 12.1) |

*LED group as reference group in analysis of covariance (ANCOVA)

*n, Number of Patients; LED, Low Energy Diet; SD, Standard Deviation; IQR, interquartile range; kg, Kilograms; m, Meters; cm, Centimeters; lbs, Pounds; SF-36, Short-Form 36; LOS, Length of Stay; mL, Millilitres; MD, Mean Difference; RD, Risk Difference; CI, Confidence Intervals*

**Supplemental Table 1.** Traffic light criteria for feasibility outcomes.

| **Variable/Outcome** | **Criteria for Success** | | |
| --- | --- | --- | --- |
|  | *Green Light* | *Yellow Light* | *Red Light* |
| *Recruitment Rate* | ≥ 16 patients per month | 10-15 patients per month | < 10 patients per month |
| *Randomization Percentage* | ≥ 70% | 50-69.9% | < 50% |
| *Intervention Adherence* | ≥ 80% | 70-79.9% | < 70% |
| *Follow-up Completion* | ≥ 90% | 80-89.9% | < 80% |
| *Network Development* | ≥ 10 | - | < 10 |

**Supplemental Table 2.** Reported reasons for non-participation

| **Reason** | **Overall**  **n=62** |
| --- | --- |
| Worried about potential adverse effects from LED (n [%]) | 9 (14.5) |
| LED protocol was too strict (n [%]) | 9 (14.5) |
| LED protocol was too long (i.e., too many weeks before surgery) (n [%]) | 3 (4.8) |
| Wanted to try and lose weight without LED (n [%]) | 6 (9.7) |
| Did not think the LED would help with clinical outcomes (n [%]) | 4 (6.5) |
| Did not want to complete research paperwork (n [%]) | 4 (6.5) |
| Did not want to pick up LED (n [%]) | 4 (6.5) |
| Did not want to come in for extra visits before surgery (n [%]) | 21 (33.9) |
| Did not want to come in for extra visits after surgery (n [%]) | 12 (19.4) |
| Live too far from study centre (n [%]) | 2 (3.2) |
| Did not want to be in the control group (n [%]) | 2 (3.2) |
| Was too overwhelmed by the idea of surgery to participate (n [%]) | 19 (30.7) |
| Travelling/have plans already during intervention period (n [%]) | 6 (9.7) |
| Other (n [%]) | 11 (17.7) |

*n, Number of Patients; LED, Low Energy Diet*

**Supplemental Table 3.** Postoperative period anthropometric outcome data

| **Outcome** | **LED**  *Baseline n=45*  *Post-LED n=45*  *Postoperative n=44* | **Control**  *Baseline n=46*  *Post-LED n=43*  *Postoperative n=42* | **MD (95% CI)*** |
| --- | --- | --- | --- |
| Weight, kg (mean [SD]) |  |  |  |
| Baseline | 107.8 (21.5) | 105.7 (18.4) | -3.2 (-4.7 to -1.7) |
| Postoperatively | 102.0 (20.6) | 104.6 (18.8) |  |
| BMI, kg/m^2^ (mean [SD]) |  |  |  |
| Baseline | 36.1 (5.6) | 36.4 (6.4) | -1.3 (-1.8 to -0.7) |
| Postoperatively | 34.1 (5.1) | 35.9 (6.7) |  |
| Waist circumference, cm (mean [SD]) |  |  |  |
| Baseline | 118.5 (13.4) | 116.3 (12.6) | -4.0 (-5.8 to -2.1) |
| Postoperatively | 112.4 (12.6) | 115.4 (14.8) |  |
| Grip strength, kg (mean [SD]) |  |  |  |
| Baseline | 30.3 (12.0) | 25.1 (9.1) | -1.2 (-3.2 to 0.7) |
| Postoperatively | 29.7 (11.5) | 26.6 (9.4) |  |

*VLED group as reference group in analysis of covariance (ANCOVA)

*n, Number of Patients; LED, Low Energy Diet; SD, Standard Deviation; kg, Kilograms; m, Meters; cm, Centimeters; MD, Mean Difference; CI, Confidence Intervals*

**Supplemental Table 4.** Overview of all randomized controlled trials evaluating VLEDs/LEDs prior to elective non-bariatric surgery.

| Author, Year | Arms | N | Inclusion | Type of Operation | Diet Details | Kg Mean Weight Loss | Postoperative Morbidity (%) |
| --- | --- | --- | --- | --- | --- | --- | --- |
| Burnand, 2016 | LED | 21 | BMI ≥ 30 | Laparoscopic Cholecystectomy | Product: Slim-Fast  Target 800 kcal/d - actual mean kcal/d 947  Breakfast and lunch 1 shake each, dinner “one less than 3% fat ready meal” | 3.5 | 1 (4.8) |
|  | Control | 25 |  |  |  | 1.0 | 1 (4.0) |
| Barth, 2019 | VLED | 30 | BMI ≥ 25 | Hepatectomy | Product: Optifast  Target 800 kcal/d - actual mean kcal/d 805  Low calorie and low-fat diet  Five units of Optifast800/d  If patients could not comply with the Optifast diet, they were instructed by study dieticians to take an alternative food diet equivalent | NR | 6 (20.0) |
|  | Control | 30 |  |  |  | NR | 5 (16.7) |
| Kip, 2019 | VLED | 4 | NR | Elective carotid endarterectomy | Product: Scandishake  Protein and calorie restriction  The Mifflin St Jeor equation employed by licensed dieticians to calculate the 24-hour energy needs of the individual participants | NR | 0 |
| Lilijensoe, 2019 | VLED | 38 | BMI ≥ 30 | Total knee replacement for OA | Product: Cambridge Weight Plan  Target 810 kcal/d  Liquid diet using commercially available formula foods  The formula diets consisted of ready-to-use meal, bars, and sachets to mix with water or skimmed milk (7.5 dL/d) to make shakes, soups, or porridge, consumed 4x/d | 10.7 | 3 (7.9) |
|  | Control | 38 |  |  |  | NR | 5 (13.1) |
| Griffin, 2024 | LED | 23 | BMI ≥ 30 | Laparoscopic  cholecystectomy, ventral hernia repair (excluding inguinal  hernia) and major gynaecology procedures | Product: Optifast, Optislim  Participants aged < 65 years were recommended to follow  ‘Phase 1 VLED’ - three VLED meal replacements  daily (800 kcal/d)  Participants aged ≥ 65  years were recommended to follow ‘Phase 2 VLED’ - meal replacements daily (about 900 kcal/d) and included  two portions of carbohydrate containing whole foods to replace | 5.2 | 4/21 (19.0) |
|  | Control | 29 |  |  |  | 0.9 | 8/22 (36.4) |
| McKechnie, 2025 | LED | 45 | BMI ≥ 30 | Intra-abdominal or lower extremity orthopedic surgery | Product: Medimeal  Patients consumed four packets of powdered Medimeal mixed with water per day, providing a total of 920 kcal/d. Additionally, they were allowed to consume up to two cups of low-calorie vegetables per day and ad-lib zero calorie fluids | 5.8 | 13/44 (29.5) |
|  | Control | 46 |  |  |  | 0.8 | 14/42 (33.3) |

*Median (range) ); †Mean (range)

*LED, Low Energy Diet; VLED, Very Low Energy Diet; N, Number of Patients; SD, Standard Deviation; Kg, Kilograms; BMI, Body Mass Index; DM, Diabetes Mellitus; T1DM, Type I Diabetes Mellitus; CVD, Cardiovascular Disease; kcal, Kilocalories; d, Day; NR, Not Reported; g, Grams; dL, Deciliter; RA, Rheumatoid Arthritis; OA, Osteoarthritis; x, Times*
