# Supplementary material for: Preoperative low-energy diets for patients with a body mass index >30 kg/m2 undergoing non-bariatric surgery: pilot feasibility randomized clinical trial and a systematic review and meta-analysis of efficacy data
Source: Br J Surg. 2026 Mar 13;113(5):znag023. doi: 10.1093/bjs/znag023 (PMC13155937; doi:10.1093/bjs/znag023)
Supplement: znag023_Supplementary_Data [file znag023_supplementary_data.zip › Supplementary_material.docx]

**
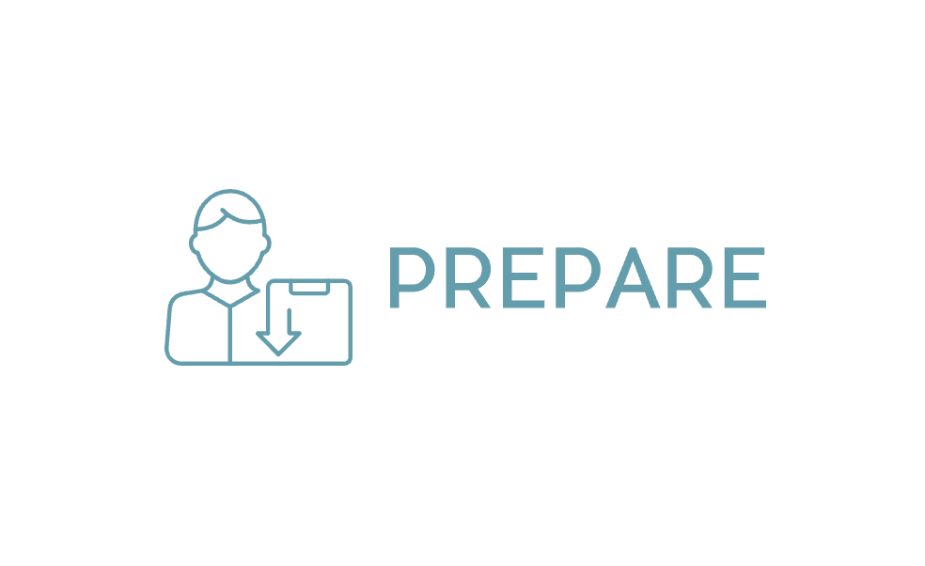
**

**PR**eoperative very low **E**nergy diets for obese **PA**tients undergoing non-bariatric surgery: A **R**andomized **E**valuation

(**PREPARE Pilot)**

**Principle Investigator**

Tyler McKechnie MD. McMaster University, Department of Surgery, Division of General Surgery, 1280 Main Street West, Hamilton, Ontario, Canada, L8S 4L8. Email: [tyler.mckechnie@medportal.ca](mailto:tyler.mckechnie@medportal.ca). Phone: (613) 868-9442.

**Supervising Investigators**

Mohit Bhandari MD, PhD, FRCSC. McMaster University, Department of Surgery, Division of Orthopedic Surgery. Hamilton General Hospital, 237 Barton Street East, Hamilton, Ontario, Canada, L8L 2X2. Email: [bhandm@mcmaster.ca](mailto:bhandm@mcmaster.ca). Phone: (905) 541-6057

Cagla Eskicioglu MD, MSc, FRCSC, FASCRS. McMaster University, Department of Surgery, Division of General Surgery. St. Joseph’s Healthcare Hamilton, 50 Charlton Avenue East, Hamilton, Ontario, Canada, L8N 4A6. Email: [eskicio@mcmaster.ca](mailto:eskicio@mcmaster.ca). Phone: (905) 522-1155 ext. 35921. Fax: (905) 540-6515.

**Co-Investigators**

Sameer Parpia PhD. McMaster University, Department of Health Research Methods, Evidence, and Impact. Email: [parpai@mcmaster.ca](mailto:parpai@mcmaster.ca)

Aristithes Doumouras MD, MPH, FRCSC. McMaster University, Department of Surgery, Division of General Surgery. Email: [Aristithes.doumouras@medportal.ca](mailto:Aristithes.doumouras@medportal.ca)

Maisa Saddik MSc. McMaster University, Department of Surgery, Division of General Surgery. Email: [saddim@mcmaster.ca](mailto:saddim@mcmaster.ca)

**TABLE OF CONTENTS**

[LIST OF ABBREVIATIONS 3](#_Toc134012101)

[STUDY SYNOPSIS 4](#_Toc134012102)

[LAY SUMMARY 7](#_Toc134012103)

[THE NEED FOR A TRIAL 8](#_Toc134012104)

[What is the problem to be addressed? 8](#_Toc134012105)

[What is/are the principal research question(s) to be addressed? 9](#_Toc134012106)

[Feasibility Objectives: 9](#_Toc134012107)

[Full Trial Objectives: 9](#_Toc134012108)

[Why is a trial needed now? 10](#_Toc134012109)

[How will the results of this trial be used? 11](#_Toc134012110)

[Are there any risks to the safety of participants involved in the trial? 12](#_Toc134012111)

[THE PROPOSED TRIAL 12](#_Toc134012112)

[What is the proposed trial design? 12](#_Toc134012113)

[What are the planned trial interventions? 12](#_Toc134012114)

[Intervention Group 12](#_Toc134012115)

[Control Group 13](#_Toc134012116)

[What are the proposed practical arrangements for allocating participants to trial groups? 13](#_Toc134012117)

[What are the proposed methods for protecting against sources of bias? 14](#_Toc134012118)

[What are the planned inclusion/exclusion criteria? 14](#_Toc134012119)

[Inclusion criteria 14](#_Toc134012120)

[Exclusion criteria 14](#_Toc134012121)

[What is the proposed duration of treatment period? 15](#_Toc134012122)

[What is the proposed frequency and duration of follow up? 15](#_Toc134012123)

[What are the proposed primary and secondary outcome measures? 16](#_Toc134012124)

[How will the outcome measures be measured at follow up? 18](#_Toc134012125)

[What is the proposed sample size and what is the justification for the assumptions underlying the power calculations? 18](#_Toc134012126)

[What is the planned recruitment rate? How will recruitment be organized? 19](#_Toc134012127)

[Are there likely to be any problems with compliance? 19](#_Toc134012128)

[What is the likely rate of loss to follow up? 19](#_Toc134012129)

[How many centers will be involved? 20](#_Toc134012130)

[What are the proposed types of analyses? 20](#_Toc134012131)

[What is the proposed frequency of analyses? 20](#_Toc134012132)

[Are there any planned subgroup analyses? 20](#_Toc134012133)

[TRIAL MANAGEMENT 20](#_Toc134012134)

[What are the arrangements for day-to-day management of the trial? 20](#_Toc134012135)

[What will be the role of each principal applicant and co-applicant proposed? 20](#_Toc134012136)

[REFERENCES 22](#_Toc134012137)

[BUDGET 30](#_Toc134012138)

[Budget justification 31](#_Toc134012139)

# LIST OF ABBREVIATIONS

| **AKI** | Acute Kidney Injury |
| --- | --- |
| **AL** | Anastomotic Leak |
| **AUR** | Acute Urinary Retention |
| **BMI** | Body Mass Index |
| **CDC** | Centers for Disease Control and Prevention |
| **CVA** | Cerebrovascular Accident |
| **DGE** | Delated Gastric Emptying |
| **DSMB** | Data Safety Monitoring |
| **dSSI** | Deep Surgical Site Infection |
| **eGFR** | Estimated Glomerular Filtration Rate |
| **Kcal** | Kilocalorie |
| **Kg** | Kilogram |
| **LOS** | Length of stay |
| **MCID** | Minimally Clinical Important Difference |
| **MI** | Myocardial Infarction |
| **MINS** | Myocardial Injury After Non-Cardiac Surgery |
| **PJI** | Prosthetic Joint Infection |
| **POI** | Prolonged Postoperative Ileus |
| **PONV** | Postoperative Nausea and Vomiting |
| **QoL** | Quality of Life |
| **RCT** | Randomized Controlled Trial |
| **SF-36** | 36-Item Short Form Survey |
| **SSI** | Surgical Site Infection |
| **sSSI** | Superficial Surgical Site Infection |
| **UTI** | Urinary Tract Infection |
| **VLED** | Very Low Energy Diet |
| **VTE** | Venous Thromboembolism |

# STUDY SYNOPSIS

| **Title** | PReoperative very low Energy diets for obese PAtients undergoing non-bariatric surgery Randomized Evaluation (PREPARE) Pilot Trial |
| --- | --- |
| **Principal Investigators** | Dr. Tyler McKechnie, Dr. Mohit Bhandari, and Dr. Cagla Eskicioglu |
| **Sponsor** | None |
| **Project Office** | St. Joseph’s Healthcare Hamilton, 50 Charlton Avenue East, Room G836, Hamilton, ON, Canada, L8N 4A6 |
| **Study Design** | Multi-center blinded, parallel pilot randomized controlled trial at Hamilton Health Sciences, St. Joseph’s Healthcare Hamilton, and Kingston Health Sciences. |
| **Study Population** | **Inclusion criteria**   - Older than 18 years of age - BMI of greater than 30 kg/m^2^ - Undergoing elective non-bariatric surgery for benign or malignant disease   **Exclusion criteria**   - Undergoing bariatric surgery (i.e., gastric bypass, gastric sleeve, duodenal switch) - Undergoing ophthalmologic or neurologic surgery - Undergoing wide local excisions for skin and/or subcutaneous lesions - Undergoing endoscopic surgery - Undergoing urgent or emergent surgery - Recently diagnosed myocardial infarction or unstable angina (i.e., within six months of assessment for trial enrolment) - Diagnosed moderate-to-severe renal dysfunction (i.e., eGFR less than 30mL/min/1.73m^2^) - Diagnosed severe liver dysfunction (i.e., cirrhosis, portal hypertension, hepatic encephalopathy, hepatorenal syndrome) - Recently diagnosed alcohol or drug use disorders (i.e., excessive use of substance within six months of assessment for trial enrolment) - Experienced a recent episode of gout (i.e., within six months of assessment for trial enrolment) - Medical history of porphyria - Known allergy to any Optifast® ingredient - Enrolled in other prospective studies with similar interventions and/or outcomes - Pregnant or breastfeeding women - Patients residing in a long-term care facility - Patients otherwise unable to provide written informed consent |
| **Sample Size** | This multicenter pilot randomized controlled trial will enrol 88 patients. |
| **Study Intervention** | The intervention being investigated is the use of Optifast® 900 or Medimeal® program for three weeks preoperatively plus preoperative standard of care |
| **Study Control** | Preoperative standard of care consisting of surgeon-delivered preoperative weight loss counselling at the time of the preoperative clinic visit |
| **Feasibility Objectives** | The feasibility objectives of this trial are:   1. Determine the feasibility of recruiting patients in a timely manner across local and outside sites. 2. Determine compliance with preoperative VLEDs in obese patients undergoing elective non-bariatric surgery. 3. Determine the feasibility of completion of follow up. 4. Determine our ability to develop a network of participating sites in a multi-site initiative. 5. Determine the safety of administering preoperative VLEDs to obese patients undergoing elective non-bariatric surgery. |
| **Full Trial Objectives** | The overall trial objectives are:   1. Determine the impact of preoperative VLEDs on postoperative morbidity in obese patients undergoing non-bariatric surgery. 2. Determine the ability for preoperative VLEDs to effectively induce significant weight loss in obese patients undergoing non-bariatric surgery. 3. Determine the effect of preoperative VLEDs on intraoperative difficulty as measured by operative time, intraoperative blood loss, and surgeon perceived difficulty. |
| **Feasibility Outcomes** | The primary outcome will be feasibility for an adequately powered randomized controlled trial. Feasibility will be measured according to the following:   1. Recruitment rate: number of patients enrolled and randomized into the trial per month. A rate of 16 patients per month (4 patients per site per month) is considered acceptable. 2. Intervention compliance: Defined as the number of doses taken will be divided by the total number of doses prescribed (84) for each participant randomized to the intervention arm. A mean compliance of greater than 80% (i.e., completing 80% or more of their VLED doses), is the benchmark for feasibility in the present study. 3. Follow-up completion: Defined as completion of patients pre-VLED, preoperative, and 30-day postoperative visits and completion of the anthropometric measures and study questionnaires. A follow-up completion rate of greater than 90% will support the feasibility of a larger RCT. |
| **Full Trial Outcomes** | The outcomes for the overall trial will be:   - Overall 30-day postoperative morbidity - 30-day system-specific complications - 30-day postoperative mortality - Preoperative weight loss - Operative time - Intraoperative blood loss - Postoperative length of stay - Quality of life |
| **Statistical Considerations for the Feasibility Study** | Feasibility Objectives: Descriptive statistics (means, medians, standard deviations, interquartile ranges, proportions) will be used to characterize the study sample. The outcomes of the pilot study are largely descriptive in nature and will focus on feasibility as opposed to statistical comparisons.  Overall Trial Objectives: We will not assess statistical significance for the overall trial clinical outcomes. However, we will calculate aggregated measures for 30-day postoperative morbidity to ensure an accurate power calculation is performed for the fully powered trial. |
| **Duration of Study Period (per patient)** | Patients will complete a course of Optifast® 900 or Medimeal® during the three weeks preceding their operative date. They will be followed throughout their index postoperative stay in hospital. They will then have follow-up at 30-days postoperatively. |

# LAY SUMMARY

Obesity has reached epidemic proportions worldwide affecting 700 million persons worldwide, representing 11% of the world’s population. The economic costs of obesity are devastating with an estimated $2.5 trillion and over $100 billion in the United States and Canada, respectively. The obese surgical patient is increasingly pervasive, which may have significant economic and clinical consequences. Obesity in the surgical setting can be extremely challenging and is associated with increased risk of both intraoperative and postoperative complications. Weight loss prior to surgery can significantly reduce these risks and contribute to enhanced postoperative recovery. Yet, preoperative optimization for obese patients has been sporadically applied and studied outside of bariatric surgery. In bariatric surgery, a surgical sub-specialty that manages obese patients almost exclusively, an intensive preoperative optimization program exists. As part of this, patients adhere to very low energy diets (VLEDs) with liquid formulations for two-to-three weeks prior to surgery in an attempt to reduce overall fat volume and improve operative conditions. Despite routine use in bariatric surgery, VLEDs have only been employed in a small number of studies evaluating obese patients undergoing non-bariatric surgery. Evidence for this low-cost initiative is promising but inconclusive. As such, we have designed a pilot randomized controlled trial (RCT) aimed at assessing the feasibility and safety of performing an adequately powered RCT for assessing efficacy of VLEDs with liquid formulation in obese patients undergoing elective non-bariatric surgery. If this pilot RCT demonstrates feasibility and safety, we will be armed with the knowledge required to pursue a larger, adequately powered, multicenter RCT with the intent of optimizing this patient population prior to elective non-bariatric surgery. Ultimately, we hope this research program can inform a preoperative optimization pathway for these patients with the aim of improving outcomes and decreasing associated healthcare burden.

# THE NEED FOR A TRIAL

## What is the problem to be addressed?

Obesity is a worldwide pandemic affecting upwards of 700 million people around the globe.^1^ The economic burden is estimated at over $2 trillion United States dollars (USD) and the resultant healthcare consequences can be devastating for systems and patients alike.^2^ Obesity adversely affects nearly all human physiologic function. It increases the risk of developing insulin resistance, cardiovascular disease, several types of cancers, mental health concerns, and more.^1,3,4^ Numerous public health campaigns have been developed with the aim of preventing these comorbidities, but these patients remain pervasive, and present significant challenges in managing acute medical and surgical conditions. Unfortunately, as of now, this problem is only set to magnify as up to 20% of children and adolescents in the United States are obese.^5^

Obese patients presenting in need of medical and surgical intervention are at higher risk of prolonged hospitalization, infectious morbidity, and venous thromboembolism.^6–8^ In the postoperative period in particular, their risk of morbidity is significantly heighted.^9^ Obese patients are at double the risk of postoperative cardiovascular, genitourinary, and wound complications as compared to their non-obese counterparts.^9,10^ Prolonged hospitalization and increased healthcare costs result.^11,12^

To pre-emptively mitigate some of these concerns prescribed preoperative weight loss via very low energy diets (VLED) is standard of care for patients undergoing bariatric surgery.^13^ These programs can effectively induce significant amounts of preoperative weight loss in these obese patients.^14–16^ This contributes to decreased postoperative length of stay (LOS), as well as decrease surgeon perceived technical difficulty.^17,18^ The impact on postoperative morbidity, however, is less clear. We performed a systematic review and meta-analysis which suggested a trend towards a significant reduction in postoperative morbidity in bariatric surgery patients, but the pooled analysis remained underpowered to adequately assess this outcome.^14^

VLEDs have only been sporadically applied and studied outside the setting of bariatric surgery.^19^ Safety and efficacy have been demonstrated in small, mostly observational studies evaluating patients undergoing both benign and malignant non-bariatric surgery, yet adequately powered prospective randomized study and the use of preoperative VLEDs in this patient population remains limited.^19^ As such, we propose to conduct a multi-center randomized controlled trial (RCT) aimed at determining the efficacy of contemporary VLEDs in obese patients undergoing a variety of non-bariatric surgeries. Ultimately, we plan on evaluating the efficacy of VLEDs as a preoperative intervention for inducing weight loss and improving perioperative outcomes via an adequately powered RCT. This is a reasonable next step given the trend towards a decrease in postoperative morbidity in bariatric surgery patients despite an underpowered analysis in addition to the sporadic study of this intervention in patients undergoing non-bariatric surgery. Prior to proceeding with a adequately powered RCT, however, a pilot RCT is warranted to confirm feasibility. Given that VLEDs are a complex intervention, assessing patient compliance with the diet and the required follow-up for data collection is crucial prior to embarking on pursuit of a multi-center international RCT.

## What is/are the principal research question(s) to be addressed?

### Feasibility Objectives:

The objective of this pilot RCT is to determine the feasibility of a multicenter, blinded RCT comparing obese patients (i.e., body mass index [BMI] greater than 30kg/m^2^) receiving VLEDs versus control prior to elective non-bariatric surgery in terms of perioperative outcomes.

The specific feasibility objectives of this trial are:

1. Determine the feasibility of recruiting patients in a timely manner across local and outside sites.
2. Determine compliance with preoperative VLEDs in obese patients undergoing elective non-bariatric surgery.
3. Determine the feasibility of completion of follow up.
4. Determine our ability to develop a network of participating sites in a multi-site initiative.
5. Determine the safety of administering preoperative VLEDs to obese patients undergoing elective non-bariatric surgery.

### Full Trial Objectives:

The objective of the fully powered multicenter, blinded RCT will be to compare obese patients receiving a VLED prior to elective non-bariatric surgery to obese patients receiving a control in terms of 30-day overall postoperative morbidity.

The specific efficacy objectives will be:

1. Determine the impact of preoperative VLEDs on postoperative morbidity in obese patients undergoing elective non-bariatric surgery.
2. Determine the ability for preoperative VLEDs to effectively induce weight loss in obese patients undergoing elective non-bariatric surgery.
3. Determine the effect of preoperative VLEDs on intraoperative difficulty as measured by operative time, intraoperative blood loss, and surgeon perceived difficulty.

## Why is a trial needed now?

Obesity is increasingly prevalent in Western society.^20^ There are over 90 million obese individuals living in the United States alone, with over half of the population projected to be obese by 2030.^21^ The obese surgical patient has become unavoidable across all surgical specialties. In abdominal surgery, these patients can present unique problems due to increased intraoperative difficulty associated with both subcutaneous and visceral fat volume.^22–25^ ^26,27^Intraoperatively, obesity is associated with increased operative time, blood loss, and surgeon perceived technical challenge.^26^ Postoperative care can present similar difficulty, as obesity is associated with increased risk of surgical site infections, urinary tract infection, pulmonary complications, wound dehiscence, venous thromboembolism, and more.^7,27,28^ Obesity may even be associated with worse long-term oncologic outcomes following resection of solid tumors.^29,30^

In bariatric surgery, a surgical sub-specialty that almost exclusively manages patients with BMIs of greater than 35 kg/m^2^, patients undergo an intensive preoperative program to optimize both intra- and postoperative outcomes. Specifically with regards to preoperative weight loss, these patients adhere to VLEDs with liquid formulations, such as Optifast® or Modifast®.^31,32^ VLEDs are an intensive approach to short-term medical weight loss that include all recommended daily micronutrients and are fortified with protein, yet are limited in their fat and carbohydrate content, allowing them to promote weight loss while maintaining lean body mass.^33–35^ ^13^These preoperative programs are safe, tolerable, and have been associated with decreased fat volume, surgeon perceived operative difficulty, operative time, and postoperative LOS in patients undergoing bariatric surgery.^18,36^ Data supporting decreased postoperative morbidity with their use in bariatric surgery patients is less convincing. We conducted a systematic review and meta-analysis evaluating the impact of preoperative VLEDs in patients undergoing bariatric surgery in terms of postoperative morbidity.^14^ Data was pooled from four RCTs and there was a non-significant trend towards decreased postoperative morbidity (OR 0.65, 95%CI 0.35-1.19, p=0.16).^14^ This analysis was underpowered despite pooling. Nonetheless, current Canadian Adult Obesity Clinical Practice Guidelines and Enhanced Recovery After Surgery (ERAS) guidelines recommend two-to-three weeks of preoperative VLED with liquid formulations totalling 650-900 kilocalories (kcal) per day.^13^

Despite the advantages associated with preoperative VLEDs in bariatric surgery, the evidence associated with and use of preoperative VLEDs in other types of surgery remains sporadic. We recently performed a systematic review and meta-analysis that identified 13 studies evaluating the use of preoperative VLEDs in non-bariatric surgery.^19^ While data were heterogenous, preoperative VLEDs reliably resulted in significant weight loss (3.2-19.2 kilograms [kg]) and were well adhered to (94-100% compliance). In the seven included comparative studies, preoperative VLEDs were not associated with changes in postoperative morbidity, postoperative LOS, or operative time. They were not associated with adverse events or intolerance in obese patients with malignancy undergoing oncologic resection. Despite seemingly being safe and well tolerated, only four, small RCTs have evaluated their use for preoperative optimization of obese non-bariatric surgery patients.^37–40^ Altogether, there are significant grounds to explore the use of preoperative VLEDs in obese patients undergoing non-bariatric surgery as a means to optimize perioperative outcomes for this increasingly pervasive patient population. Moreover, our data pertaining to postoperative morbidity in bariatric surgery patients adds clinical equipoise to this patient-important outcome.

The current evidence base for preoperative VLEDs for obese patients prior to non-bariatric surgery is far from conclusive. Surgeon practice across the country is highly variable as a result. We are currently conducting a Canada-wide survey targeted at assessing current practice patterns in terms of preoperative weight loss interventions for obese patients undergoing non-bariatric abdominal surgery. Preliminary data suggest that most surgeons do not prescribe preoperative weight loss interventions to these patients, nor do they feel as though they foundational knowledge of the preoperative weight loss interventions currently available. Given the lack of high-quality evidence currently informing the use of preoperative VLEDs in this increasingly pervasive, and challenging, patient population, there is currently a highly justified basis for a large, high-quality, prospective trial to be conducted in this field. We have assembled a committed community of surgeons and solid trial infrastructure to pursue this endeavour and execute a large, definitive RCT aimed at assessing the efficacy of preoperative VLEDs for obese patients undergoing non-bariatric surgery.

## How will the results of this trial be used?

The feasibility and safety results of this pilot RCT will be used to inform the design and implementation of an adequately powered multi-center RCT aimed at assessing the efficacy of VLEDs at improving perioperative outcomes for obese patients undergoing non-bariatric surgery. We will use our pilot RCT as a platform to build further awareness about this omnipresent issue in contemporary surgery with the hope of recruiting physicians from other centers to participate in our definitive RCT. Ultimately, these data will inform the development of standardized and individualized preoperative optimization pathways for patients undergoing non-bariatric surgery for benign and malignant disease.

Both the findings of this pilot RCT as well as the overall trial will be published in peer reviewed medical journals and presented at international, national, and local conferences as part of our knowledge translation strategy. We will use these findings, in addition to other pertinent literature, to advocate for the creation of preoperative optimization guidelines for patients undergoing major surgery. We will liaise with large societies such as the Canadian Association of General Surgeons, Society of American Gastrointestinal and Endoscopic Surgeons, American Society of Colon and Rectum Surgeons, Enhanced Recovery After Surgery Society, and more for the development and dissemination of these guidelines. We have access to online platforms with reach spanning to surgeons across the world, such as OrthoEvidence and Cold Steele, that we will rely on to widely circulate these findings. Lastly, we will work with industry sponsors to create community outreach programs aimed at providing smaller, rural committees access to preoperative VLED products with the aim of enhancing preoperative optimization for obese patients undergoing surgery in all communities around the world.

## Are there any risks to the safety of participants involved in the trial?

Patients enrolled in this RCT will be randomized to receive either standard of care plus Optifast®/Medimeal® or standard of care alone. The standard of care arm would not be incurring incremental risk, though patients randomized to Optifast®/Medimeal® are at risk of mild and severe adverse events. Adverse events are listed in a separate document titled “Adverse Events”. The largest (n = 273) medical weight loss RCT utilizing Optifast® to date reports a mild adverse event rate of 76.8% and serious adverse event rate of 4.5% in patients enrolled in a yearlong program.^41^ The most commonly reported mild adverse events included constipation (18.7%), headache (17.4%) and dizziness (16.8%).^41^ Serious adverse events included symptomatic cholelithiasis, pyelonephritis, and seventh cranial nerve palsy.^41^ These data are most relevant to the long-term use of Optifast® and other similar VLEDs. In our systematic review the median intervention length was four weeks.^19^ Only one included study reported the occurrence of serious adverse events, at a rate of 7.8% over an eight-week intervention period.^37^ The remaining 12 studies in the systematic review, six of which included cancer patients, did not report serious adverse events related to liquid formulation weight loss products. Minor adverse events occurred in 0-50% of patients in the included studies, with only two studies reporting rates of greater than 14%.^37,42^ Both of these studies implemented VLED programs spanning eight weeks. Overall, while there are risks associated with VLED use, these risks are mostly limited to minor adverse events when used in the preoperative setting and we are confident that it will be a safe intervention for patients meeting inclusion criteria. Particularly given that the proposed duration of VLED in the present study is three weeks. There are no interactions with Optifast®/Medimeal® and prescribed medications that may put patients at risk. Overall, the risk associated with the use of Optifast®/Medimeal® in this trial should be minimal given that this product is already approved for this indication in this patient population.

# THE PROPOSED TRIAL

## What is the proposed trial design?

The proposed trial will be a multi-center, surgeon, outcome assessor, and data-analyst blinded, parallel pilot RCT.

## What are the planned trial interventions?

### Intervention Group

The preoperative VLED protocol will utilize Optifast® or Medimeal®, commercially available weight loss products. Specifically, the Optifast® 900 product will be used for this RCT. Patients will receive a three-week supply of Optifast® 900 or Medimeal®. They will be instructed to consume four packets of Optifast® 900 or Medimeal® mixed with water daily. This provides a total energy intake of 900 kcal per day. Patients will also be allowed to consume up to two-cups of low-calorie vegetables per day along with the Optifast® meal replacement product. They will be provided with a handout containing specific instructions with regards to the intervention (see “Very Low Energy Diet Instructions” document). The intervention group will receive the Optifast® 900 program or Medimeal®, as described above, in addition to standard of care for preoperative management.

Patients enrolled in the trial and assigned to the VLED group who are diabetics will be referred to a diabetes educator nurse practitioner for management of their insulin dosing and/or other anti-hyperglycemic medications throughout the duration of the intervention period.

### Control Group

The control group will consist of standard of care for preoperative management of obese patients undergoing elective non-bariatric surgery without prescription of a specific preoperative weight loss intervention. Currently, there are no standardized interventions aimed at optimizing obese patients prior to undergoing non-bariatric surgery. These patients will routinely receive counselling from their surgeon at the time of their preoperative clinic visit targeted towards preoperative weight loss recommendations. Recommendations are not standardized, nor evidence based. There is no intervention lasting beyond counseling at this visit. Patients will not receive prescriptions for preoperative VLEDs, any other weight loss supplement or medication, or any physical activity intervention aimed at promoting weight loss prior to surgery.

## What are the proposed practical arrangements for allocating participants to trial groups?

Patient recruitment will occur at Hamilton Health Sciences, St. Joseph’s Healthcare Hamilton, and Kingston Health Sciences. All surgeons who perform elective non-bariatric surgery at these sites will be made aware of the study via workshops and internal communications. Patients will be identified by the surgeons, their administrative assistants, and/or study investigators at the time of referral, at the time of their initial preoperative consultation, or some time following their initial preoperative consultation. Surgeons and/or other clinicians within a patient’s circle of care will introduce the study to participants at the time of their preoperative visits or sometime thereafter. Patients may also reach out directly to study investigators via the contact information provided on the study poster should they choose. Patients meeting inclusion criteria based on a review of their electronic medical record, which will only be accessed by persons within the circle of care or researchers that have obtained permission from the patient, will have their contact information (i.e., email and phone number) recorded and provided to designated research personnel at the time of their initial surgical consultation in clinic. This email contact information will then be passed into a novel automated clinical trials platform developed by Phelix AI©. This platform will automatically send an electronic version of the written informed consent document to the patient as well as a link to schedule an appointment in the following 1-2 days with the research team to discuss the informed consent document via telephone. If the Phelix AI© platform identifies that the patient does not have an accessible email address, this will trigger a notification to the research team and prompt them to call the patient by telephone. At the time of initial contact between the study personnel and patient, the study will be introduced, eligibility will be confirmed. Patients will be provided with the opportunity to ask questions about the previously circulated ICF, and if they have yet to review the document, they will review the document in its entirety with the research team at the time of the telephone contact. If, at the end of this process, patients are understanding of the material risks and benefits of participation, and wish to be enrolled in the study, they will sign the ICF and subsequently be randomized to one of the two arms in this parallel RCT. Following ascertainment of written informed consent, patients will be randomized via a web-based randomization system (randomization.net) in a 1:1 fashion to receive either standard of care plus preoperative VLED or standard of care alone. Randomization will be according to variable block sizes of four and six and will be stratified according to centre.

Individuals with literacy, visual, and hearing issues will be included in the present study. As a research team, we will ensure that the ICF is written in lay language to a level that is understood by the majority of participants. For potential participants who have literacy issues, we will review the ICF and any other study-related questions in detail with them and any family member(s) or friend(s) with whom they would like to review this information with. If the participant demonstrates an understanding of the study following this review, they will be eligible to participate should they voluntarily choose to. For potential participants with visual issues, the ICF will be reviewed verbally with them in detail by a member of the study team in the presence of any family member(s) or friend(s) that the patient chooses. If the participant demonstrates an understanding of the study following this review, they will be eligible to participate should they voluntarily choose to. Lastly, for potential participants with hearing issues, the ICF will be given to them in written format and they will have the opportunity to review the form with the research team and their family member(s)/friend(s). If the participant demonstrates an understanding of the study following this review, they will be eligible to participate should they voluntarily choose to.

The aforementioned automated clinical trials platform developed by Phelix AI© is a novel platform that automates the “patient research journey”. The research team will input the patient contact information into the Phelix AI© platform and the platform will then automatically start patients along their journey. The specific tasks that this platform will perform for the purposes of this trial include: 1) sending the ICF document; 2) scheduling a telephone follow-up with researchers to discuss the ICF document and complete the informed consent process; 3) send reminders to patients at the 1-week and 2-week points of the intervention period to remind patients to adhere to the study protocol; 4) schedule a post-intervention follow-up appointment with the research team; 5) send a follow-up email to patients two weeks after their scheduled surgery to ascertain whether any adverse events have occurred and whether they have required any care outside of the hospital in which they underwent their index operation; 6) schedule the 30-day postoperative visit with the research team. Neither this platform nor the company will retain or store any patient or research information asides from the patient email address for contact throughout the trial. Phelix’s network and services are cloud native (i.e., built on and benefit from the security measures provided by Google’s Cloud Platform (GCP)). Phelix classifies email address as Personal Data. All data, including Personal Data, is at all times hosted on Canadian servers and resources on Google Cloud Platform. Phelix does not access, share or process Personal Data beyond what is minimally required to enable customers access their Phelix services.  Email addresses used by your organization in its engagement with Phelix are not shared externally, and only shared internally on a need-to-know basis among the Phelix personnel (i.e., the researchers) with whom you will interact by email. Otherwise, at all times, whether in transit or at rest, email addresses are treated as any other Personal Data stored on Phelix, and will be anonymized, encrypted, and securely hosted.

The technology used to design this platform has been ported from the healthcare administrative platform that Phelix AI© has developed. This is widely used across the United States and Canada to handle personal health information in a safe and efficient way alongside electronic medical records such as Oscar Pro and Accuro. Namely, this technology has been used to triage referrals, pull information from faxed consultations, schedule appointments, schedule investigations, and more. Further information on Phelix AI© and their health administrative platform can be found on their website: <https://www.phelix.ai/>.

## What are the proposed methods for protecting against sources of bias?

This trial will be randomized with variable block sizes which will protect against selection bias. In addition, surgeons, outcome assessors, and data analysts will be blinded to treatment allocation in order to reduce the risk of selection bias, measurement bias, and detection bias. Blinding of outcome assessors will be achieved via the establishment of an Independent Outcomes Adjudication committee. Given the nature of the intervention, we will be unable to blind patients which may raise the possibility of recall bias during reporting of symptom and quality of life questionnaires at the one-month follow-up. We will aim for completeness of follow-up in all randomized patients which will protect against imbalance of prognosis afforded by our randomization strategy. Lastly, we will limit crossovers and co-interventions through thorough documentation of the preoperative period to avoid contamination and the resultant impact on effect size estimates.

## What are the planned inclusion/exclusion criteria?

### Inclusion criteria

- Older than 18 years of age
- BMI of greater than 30 kg/m^2^
- Undergoing major elective non-bariatric surgery*.* Major surgery is defined as any operation performed under general anesthesia requiring a skin incision extending beyond the subcutaneous tissue.

### Exclusion criteria

- Undergoing bariatric surgery (i.e., gastric bypass, gastric sleeve, duodenal switch)
- Undergoing ophthalmologic or neurologic surgery
- Undergoing wide local excisions for skin and/or subcutaneous lesions
- Undergoing endoscopic surgery
- Undergoing urgent or emergent surgery
- Recently diagnosed myocardial infarction or unstable angina (i.e., within six months of assessment for trial enrolment)
- Diagnosed moderate-to-severe renal dysfunction (i.e., eGFR less than 30mL/min/1.73m^2^)
- Diagnosed severe liver dysfunction (i.e., cirrhosis, portal hypertension, hepatic encephalopathy, hepatorenal syndrome)
- Recently diagnosed alcohol or drug use disorders (i.e., excessive use of substance within six months of assessment for trial enrolment)
- Experienced a recent episode of gout (i.e., within six months of assessment for trial enrolment)
- Medical history of porphyria
- Known allergy to any Optifast® or Medimeal® ingredient (see “Optifast 900 Program” and “Medimeal Program” document)
- Enrolled in other prospective studies with similar interventions and/or outcomes. Co-enrollment may be deemed appropriate if the steering committees of the respective trials review the details of participation and agree that the studies and their interventions should not impact one another. Participation in non-interventional studies will be allowed.
- Pregnant or breastfeeding women
- Patients residing in a long-term care facility
- Patients otherwise unable to provide written informed consent

## What is the proposed duration of treatment period?

The current protocol in use at our local Bariatric Surgery Center of Excellence (i.e., St. Joseph’s Healthcare Hamilton) utilizes four doses of Optifast® 900 or Medimeal® daily and the total length varies between one and three weeks based on preoperative liver size and BMI. To standardize the approach for non-bariatric surgery, we propose an intervention period of three-weeks. Patients will start the intervention period three weeks and one day prior to their operative date, and the last day of the intervention will be the day prior to surgery. Our systematic review identified protocols varying from 0.46-26 weeks that were safe and efficacious, and thus we are confident that our intervention period is appropriate.^19^ Similarly, the current protocol in use at our local Bariatric Surgery Center of Excellence is in keeping with the findings of our systematic review in terms of VLED liquid formulation product and caloric intake target. In our systematic review and meta-analysis, Optifast® was the most commonly studied VLED liquid formulation (35.7% of included studies) and daily target caloric intake ranged from 450-1400kcal per day.^19^ As mentioned previously, the present study will also use the Optifast® 900 or Medimeal® and will aim to provide a total of approximately 900 kcal per day to participants.

## What is the proposed frequency and duration of follow up?

Patients will attend follow up with their respective surgeons as per the surgeon’s clinical follow-up schedule for the given procedure. From a research perspective, the patient follow-up protocol will be implemented following initiation of the VLED. See Figure 1 for the SPIRIT diagram depicting the research follow-up protocol. Patients will be followed for a period of approximately 8 weeks. Patients will have in-person baseline measures, including weight, height, waist circumference, hand grip strength, and symptom screening at the time of preoperative VLED initiation, the day of their surgery, and at 30-days following the date of their surgery. On the day of their surgery, patient data will also be collected with regards to compliance with the preoperative VLED. At the one-month follow-up, patients will also complete standardized follow-up questionnaires that will focus on adverse events, symptoms, and postoperative recovery, as well as the 36-Item Short Form Survey (SF-36). Electronic medical records will be reviewed at thirty-days postoperatively to assess for postoperative morbidity and mortality. Both the clinical and research follow-up will be consistent regardless of the arm the patient is allocated to.

| **Activity** | **Before Randomization** | **-23** | **After Randomization** | | | |
| --- | --- | --- | --- | --- | --- | --- |
|  | **-60 to -23** |  | **-23 to -2** | **-2 to 0** | **0 to discharge** | **+30** |
| Eligibility assessment | X |  |  |  |  |  |
| Informed consent | X |  |  |  |  |  |
| Randomization |  | X |  |  |  |  |
| Intervention (i.e., VLED) |  |  | X |  |  |  |
| Baseline demographic data | X |  |  |  |  |  |
| Operative data collection |  |  |  | X |  |  |
| Baseline anthropometrics (including hand grip strength) | X |  |  |  |  |  |
| Post-intervention anthropometrics |  |  |  | X |  |  |
| Post-surgical anthropometrics |  |  |  |  |  | X |
| Intervention-associated adverse events |  |  | X | X | X | X |
| Postoperative morbidity |  |  |  |  | X | X |
| Surgeon perceived difficulty |  |  |  | X |  |  |
| QoL assessment (i.e., SF-36) | X |  |  | X |  | X |
| Intervention adherence |  |  | X | X |  |  |
| Follow-up completion |  |  |  |  |  | X |

*Figure 1.* SPIRIT figure for participant timeline

*VLED, very low energy diet; QoL, quality of life*

## What are the proposed primary and secondary outcome measures?

Detailed criteria for event adjudication are reported in the associated documented titled “Trial Outcome Definitions”. An outcome adjudication committee will be formulated who will independently identify and report outcomes. This committee will be blinded to treatment allocation. Given that this is a pilot trial, the primary outcome will be feasibility.

Feasibility Outcomes:

1. Randomization percentage: Defined as the number of patients agreeing to participate in the RCT and being randomized to treatment or control divided by the number of patients approached for participation in the RCT. A randomization percentage of 70% or greater will support the feasibility of a full RCT. A lesser randomization percentage may be feasible with modifications.
2. Recruitment rate: Defined as the number of patients enrolled and randomized into the RCT per month. We will aim for a rate of 16 patients per month (i.e., 4 patients per site per month). A recruitment rate of equal to or greater than this will support the feasibility of a fully powered RCT. A recruitment rate of greater than 10 but less than 16 patients per month will be considered feasible with modifications for the larger RCT. A recruitment rate of less than 10 patients per month will suggest that a larger RCT is not feasible. For the full RCT, we anticipate participation of at least 10 sites. If each site recruited 4 patients per month, this would equate to 48 patients per year. Therefore, after 2.5 years of recruitment, we would reach our pre-specified sample size of approximately 1,200 patients.
3. Intervention compliance: Defined as the number of preoperative VLED doses taken divided by the total number of doses prescribed (84) for each participant randomized to the intervention arm. Compliance will be self-reported via written diet diaries (see “Study Diet Diary” document). A mean compliance of greater than 80% (i.e., completing 80% or more of their Optifast® 900 or Medimeal® doses), will be our benchmark for feasibility in the present study. A mean compliance between 70% and 80% will be considered feasible with modifications for the larger RCT. A mean compliance of less than 70% will suggest that a larger RCT is not feasible.
4. Follow-up completion: Defined as completion of the pre-VLED, preoperative, and thirty-day postoperative visits, along with complete anthropometric measures and study questionnaires. A follow-up completion rate of greater than 90% will support the feasibility of a larger RCT. A follow-up completion rate of 80-90% will be considered feasible with modifications for the adequately powered RCT. A follow-up completion rate of less than 80% will suggest that a larger RCT is not feasible.

Safety Outcomes:

The primary safety outcome will be adverse events deemed secondary to the Optifast® 900 or Medimeal® programs. The adverse events will be recorded as dichotomous outcomes and described as either minor or serious, in a similar fashion to the OPTIWIN Study; the largest medical weight loss RCT evaluating Optifast®.^41^ Standardized definitions for minor and major adverse events are described in “Adverse Events” associated document. We will also measure patient frailty with grip strength at the time of anthropometric measurement.^43^

Clinical Outcomes:

The efficacy outcomes will include:

1. Overall 30-day postoperative morbidity. This will be defined as any deviation from the usual postoperative course within 30-days of the index operation and will be a composite of system-specific complications (see “Trial Outcome Definitions” document)
2. 30-day system-specific complications (see “Trial Outcome Definitions” document)
3. 30-day postoperative mortality
4. Preoperative weight loss. Preoperative weight loss will be assessed by measuring the post-VLED weight on the date of surgery and adjusting for the baseline weight in kilograms, as measured in a standardized fashion by a blinded research assistant.
5. Operative time. Operative time was chosen as an outcome as it is a commonly used objective surrogate for operative difficulty across various types of surgery.^44–46^ It has been used in previous bariatric RCTs as the primary outcome.^47^ Operative time will be measured as the time between first skin incision and closure of the last surgical wound in minutes and will be retrieved from the patient electronic medical record.
6. Intraoperative blood loss. This will be measured in milliliters and will be ascertained from the patient chart.
7. Postoperative length of stay (LOS). This will be measured in days and will be ascertained from the patient chart. Postoperative LOS will be calculated as the number of days following the index surgery (i.e., postoperative day 0) that the patient remains in hospital. Every morning that the patient remains in hospital will count as an added day to postoperative LOS.
8. Quality of life. This will be assessed at baseline, following completion of the VLED, and 30-days postoperatively using the SF-36 (see associated “SF-36” document) administered by a blinded research assistant. The SF-36 has been validated in numerous previous cohorts of patients undergoing both non-bariatric surgery.^48,49^

## How will the outcome measures be measured at follow up?

The feasibility measures will be ascertained by the number of patients recruited and the number of patients completing follow-up as recorded on a standardized form by a research assistant, as well as patient reported compliance via a patient-completed diet diary that they will complete daily during the three-week period prior to surgery. Both the treatment and control arms will complete these forms and give them to the research assistant in an opaque envelope on the date of their surgery. Patients in the control arms will be instructed to leave the form blank.

Safety data will be ascertained via a combination of patient self-reporting and electronic medical record data. Paper and/or electronic questionnaires will be used to assess the presence of adverse events at baseline, the morning prior to surgery, and at 30-days postoperatively.

Similarly, efficacy data will be ascertained via review of the electronic medical record. Electronic medical records will be reviewed on the date of surgery and at 30-days postoperatively to assess for any further adverse events and to evaluate 30-day postoperative morbidity. We will assess operative time, intraoperative blood loss, and postoperative length of stay via electronic medical records. The quality-of-life data will be ascertained at baseline, following completion of the VLED, and at 30-days postoperatively via administration of an in-person paper or electronic SF-36 questionnaire. Lastly, anthropometric data (i.e., height, weight, waist circumference, grip strength) will be measured at baseline, the morning of surgery, and at 30-days postoperatively by a research assistant blinded to treatment allocation. This will be recorded and stored via a Redcap© form created *a priori*.

## What is the proposed sample size and what is the justification for the assumptions underlying the power calculations?

We propose a pilot RCT of 88 patients (i.e., 22 patients per site) to assess feasibility of a full RCT aimed at determining the efficacy of preoperative VLED at improving short-term postoperative outcomes in obese patients undergoing non-bariatric surgery. This sample size is sufficient to ascertain our feasibility objectives and is approximately 7.5% of the size of a definitive trial. A full RCT would require 1,158 patients (“Full Trial Power Calculation” document). We justified our proposed sample size of 88 patients based on 95% CIs for one of our feasibility outcomes. Specifically, to assess for 90% follow-up completion, 88 patients would provide 95% CIs of 82-95%, which we believe is adequate precision.

## What is the planned recruitment rate? How will recruitment be organized?

The anticipated recruitment rate will be 16 patients per month. Patient enrollment will continue until 88 patients are enrolled (i.e., approximately 5.5 months). There are over 200 elective non-bariatric abdominal surgeries performed monthly across the four included sites, and approximately 26.6% of Canadians are obese, which equates to approximately 53 patients per month that may be potentially eligible for enrollment.^50^ Moreover, there are over 400 elective orthopedic surgeries performed monthly across the four included sites, and given the same prevalence of obesity as above, this would equate to another approximate 106 obese patients per month that may be potentially eligible for enrollment.^50^ There are over 200 cardiac surgeries performed per month at our cardiac surgery center, equating to another 53 potentially obese patients eligible per month. Across Otolaryngology, Plastic Surgery, and Neurosurgery, there are at least another 200 elective cases performed per month, thus another 53 potentially eligible obese patients per month. Altogether, there will likely be over 250 potentially eligible patients per month.

All surgeons who perform elective non-bariatric surgery will be asked to consent to allow their patients to be approached. Patients will be identified from outpatient clinic schedules via the institution’s (i.e., Epic) or surgeon’s (e.g., Oscar) electronic medical record. Upon review of their chart, should the patient meet inclusion criteria and not meet any apparent exclusion criteria, they will be approached by research personnel who will provide further details with regards to the RCT. Recruitment will be organized and implemented by Dr. Tyler McKechnie and the research assistant(s) under the guidance of the coordinating center and steering committee.

## Are there likely to be any problems with compliance?

Adherence to a preoperative two-to-three-week regimen of preoperative VLED prior to bariatric surgery ranges from 80-90%.^51,52^ In our systematic review and meta-analysis evaluating the use of VLEDs in non-bariatric surgery, reported adherence ranged from 94-100% amongst the included studies, with much longer durations of intervention (0.46-24 weeks).^19^ As such, we feel as though compliance in the present study should be similar and is anticipated to range between 80% and 100%. In order to increase adherence with the study intervention, we will cover the cost of the Optifast® 900 or Medimeal® program and have patients complete a diet diary that they will submit following the three-week intervention period.

## What is the likely rate of loss to follow up?

We don’t anticipate a significant proportion of patients lost to follow-up in the present study given the small sample size, the short-term follow-up of one-month, and the lack of complicated and taxing study protocol for patients to adhere to. The aim of the present study to demonstrate feasibility is a study follow-up completion rate of greater than 90%. A follow-up completion rate of 80-90% will be considered feasible with modifications for the adequately powered RCT. A follow-up completion rate of less than 80% will suggest that a larger RCT is not feasible.

## How many centers will be involved?

This pilot trial will include four centers: 1) St. Joseph’s Healthcare Hamilton; 2) Juravinski Hospital; 3) Hamilton General Hospital; 4) Kingston Health Sciences.

## What are the proposed types of analyses?

Descriptive statistics (means, medians, standard deviations, interquartile ranges, proportions) will be used to characterize the study sample. The outcomes of the pilot study will be descriptive in nature and will focus on feasibility. Binary outcomes will be described using proportions with corresponding 95% CIs estimated using the Wilson Score method. Differences in continuous outcomes will be described using the difference in means with corresponding 95% CIs. Considering the feasibility nature of this pilot study, and following the 2010 CONSORT statement, we will not assess statistical significance for outcomes.^53^ We will calculate aggregated measures for 30-day postoperative morbidity to compare this to the tentative power calculation in the “Full Trial Power Calculation” associated document, to ensure an accurate power calculation is performed for the fully powered RCT. The proposed data analysis plan for the full RCT is reported in the “Full Trial Statistical Analysis Plan” associated document. Data will be analyzed using STATA statistical software (StataCorp version 15; College Station, TX). All data analysis will be completed by a blinded statistician.

## What is the proposed frequency of analyses?

There will not be a planned interim analysis in the present study given that it is a pilot study with a small anticipated sample size (n=88).

## Are there any planned subgroup analyses?

There are no subgroup analyses planned as this is a pilot RCT.^54^

# TRIAL MANAGEMENT

## What are the arrangements for day-to-day management of the trial?

Patient recruitment will occur through individual surgeon clinics as well as the clinic of one of the principal investigators (Dr. Eskicioglu), should extra visits be required. Data will be captured via a combination of anthropometric measurements (i.e., height, weight), patient completed paper/electronic forms and questionnaires, and electronic medical records. De-identified data will be recorded in a firewall, password protected database. All study records will be kept on an encrypted and password-protected computer that will be kept with our lead biostatistician (Dr. Parpia). Only authorized study personnel will have access to these data.

## What will be the role of each principal applicant and co-applicant proposed?

Dr. Tyler McKechnie is a third-year General Surgery resident currently enrolled in the Clinical Investigator Program completing a thesis-based Master’s in Clinical Epidemiology through McMaster University’s Department of Health Research Methods, Evidence, and Impact. He will be the primary investigator for this project and will lead trial design, grant submissions, research ethics board application, management of research assistants, patient recruitment, day-to-day management of the trial, manuscript preparation, and knowledge translation. This project will serve as his thesis project for his Master’s Degree. Dr. Mohit Bhandari is a Distinguished McMaster University Professor and is the current Chair of the Department of Surgery. He has extensive experience conducting large, international RCTs, as well as a strong track record in overseeing and supporting graduate students. He will oversee the development and conduct of the present RCT as one of two principal supervising investigators. Dr. Cagla Eskicioglu is an academic colorectal surgeon with a Master’s Degree in Clinical Epidemiology. She will be a principal supervising investigator and will bring both clinical and methodological expertise. Dr. Sameer Parpia is a PhD biostatistician with extensive background in surgical RCTs and will be providing statistical support for the trial. Dr. Aristithes Doumouras is an academic bariatric surgeon with a Master’s in public health and personal experience with conducting surgical RCTs. He will provide clinical, methodological, and statistical expertise. Ms. Maisa Saddik is a research assistant with the Department of Surgery at McMaster University. She has graduate training in epidemiology and will in the daily activities of the trial.

**Describe the trial steering committee and if relevant the data safety and monitoring committee.**

Dr. Tyler McKechnie will be the principal investigator for the present RCT, and part of his responsibilities will be to oversee day-to-day trial management. He will be supported by a steering committee, as well as research assistants and biostatisticians. The steering committee will be involved through study inception until study completion. The steering committee will oversee design, data collection, data management, interpretation of the data, and manuscript generation. This committee will hold the primary responsibility for publication of the trial results. It will be comprised of both clinical and methodologic experts; specifically, the principal investigators, national leaders, and individuals with knowledge in specific areas or in clinical trial methods. Dr. McKechnie will meet with the steering committee at least once during the planning, recruitment, and close-out phases of the trial, and at any other time point as deemed necessary by the principal investigators.

To provide more frequent oversight of the study, a subset of the steering committee will constitute the operations committee. This committee will be composed of a select group of investigators chosen for their specific expertise and experience. The operations committee is ultimately responsible for all decisions regarding the design and conduct of the trial. Outcome adjudication committees and statistical analysis sub-committees blinded to treatment allocation will be formed and oversee data collection, extraction, and analysis. An equity, diversity, and inclusion (EDI) sub-committee will be formed to ensure that all relevant EDI aspects of this trial are identified and managed.

An independent Data Monitoring Committee (DMC) will be established and lead by an independent physician to monitor and adjudicate adverse events throughout the trial. Occurrence of all serious adverse events will be collected, where applicable, in keeping with laws and regulations governing the study site. All serious adverse events will be fully documented on appropriate case report forms outlining onset, duration, treatment required, outcome, and action taken. These data will be iteratively evaluated by the DMC and should there be cause for concern for patient safety moving forward at any point in the trial, they will consult with the principal investigators to discuss premature termination of the study.

# REFERENCES

1. Chooi YC, Ding C, Magkos F. The epidemiology of obesity. *Metabolism*. 2019;92:6-10. doi:10.1016/j.metabol.2018.09.005

2. Tremmel M, Gerdtham UG, Nilsson PM, Saha S. Economic burden of obesity: A systematic literature review. *Int J Environ Res Public Health*. 2017;14(4). doi:10.3390/ijerph14040435

3. Bianchini F, Kaaks R, Vainio H. Review Overweight , obesity , and cancer risk. 2002;3(September):565-574.

4. Mokdad AH, Ford ES, Bowman BA, et al. Prevalence of Obesity, Diabetes, and Obesity-Related Health Risk Factors, 2001. *JAMA*. 2003;289(1):76-79. doi:10.1001/jama.289.1.76

5. Bryan S, Afful J, Carroll M, et al. *National Health and Nutrition Examination Survey 2017–March 2020 Pre-Pandemic Data Files*.; 2021. doi:10.15620/cdc:106273

6. Stein PD, Beemath A, Olson RE. Obesity as a risk factor in venous thromboembolism. *Am J Med*. 2005;118(9):978-980. doi:10.1016/j.amjmed.2005.03.012

7. Tjeertes EEKM, Hoeks SSE, Beks SSBJC, Valentijn TTM, Hoofwijk AAGM, Stolker RJRJ. Obesity - a risk factor for postoperative complications in general surgery? *BMC Anesthesiol*. 2015;15(1):1-7. doi:10.1186/s12871-015-0096-7

8. Pasulka P, Bistrian B, Benotti P, Blackburn G. The Risks of Surgery in Obese Patients. *Ann Intern Med*. 1986;104(4):540-546. doi:10.7326/0003-4819-104-4-540

9. Bamgbade OA, Rutter TW, Nafiu OO, Dorje P. Postoperative complications in obese and nonobese patients. *World J Surg*. 2007;31(3):556-560. doi:10.1007/s00268-006-0305-0

10. Doyle SL, Lysaght J, Reynolds J v. Obesity and post-operative complications in patients undergoing non-bariatric surgery. *Obesity Reviews*. 2010;11(12):875-886. doi:10.1111/j.1467-789X.2009.00700.x

11. Rocha AT, de Vasconcellos AG, da Luz Neto E, Araujo AM, Alves ES, Lopes AA. Risk of Venous Thromboembolism and Efficacy of Thromboprophylaxis in Hospitalized Obese Medical Patients and in Obese Patients Undergoing Bariatric Surgery. *Obes Surg*. 2006;16:1645-1655.

12. Pickkers P, de Keizer N, Dusseljee J, Weerheijm D, van der Hoeven JG, Peek N. Body mass index is associated with hospital mortality in Critically III patients: An observational cohort study. *Crit Care Med*. 2013;41(8):1878-1883. doi:10.1097/CCM.0b013e31828a2aa1

13. Glazer S, Biertho L. Bariatric Surgery: Selection & Preoperative Workup . Canadian Adult Obesity Clinical Practice Guidelines. Published 2020. Accessed October 19, 2022. https://obesitycanada.ca/wp-content/uploads/2020/08/Bariatric-Surgery.pdf

14. McKechnie T, Lee Y, Doumouras A, Parpia S, Bhandari M, Eskicioglu C. The Impact of Very Low Energy Diets Prior to Bariatric Surgery on Postoperative Morbidity: A Systematic Review and Meta-Analysis. Published online 2023.

15. Cassie S, Menezes C, Birch DW, Shi X, Karmali S. Effect of preoperative weight loss in bariatric surgical patients: A systematic review. *Surgery for Obesity and Related Diseases*. 2011;7(6):760-767. doi:10.1016/j.soard.2011.08.011

16. Van Nieuwenhove Y, Dambrauskas Z, Campillo-Soto A, et al. *Preoperative Very Low-Calorie Diet and Operative Outcome After Laparoscopic Gastric Bypass A Randomized Multicenter Study*.

17. van Nieuwenhove Y, Dambrauskas Z, Campillo-Soto A, et al. Preoperative Very Low-Calorie Diet and Operative Outcome After Laparoscopic Gastric Bypass A Randomized Multicenter Study. *Arch Surg*. 2011;146(11):1300-1305.

18. Hutcheon DA, Hale AL, Ewing JA, et al. Short-Term Preoperative Weight Loss and Postoperative Outcomes in Bariatric Surgery. *J Am Coll Surg*. 2018;226(4):514-524. doi:10.1016/j.jamcollsurg.2017.12.032

19. McKechnie T, Povolo CA, Lee J, et al. Very Low Energy Diets Prior to Non-Bariatric Surgery: A Systematic Review and Meta-Analysis. *Surgery*. 2022;In Press.

20. WHO. *Obesity and Overweight*.; 2016. Accessed October 31, 2019. http://www.who.int/mediacentre/factsheets/fs311/ en/2016

21. Ward ZJ, Bleich SN, Cradock AL, et al. Projected U.S. State-Level Prevalence of Adult Obesity and Severe Obesity. *New England Journal of Medicine*. 2019;381(25):2440-2450. doi:10.1056/NEJMsa1909301

22. Bocca G, Mastoridis S, Yeung T, James DRC, Cunningham C. Visceral-to-subcutaneous fat ratio exhibits strongest association with early post-operative outcomes in patients undergoing surgery for advanced rectal cancer. *Int J Colorectal Dis*. 2022;37(8):1893-1900. doi:10.1007/s00384-022-04221-8

23. Collins J, McCloskey C, Titchner R, et al. Preoperative weight loss in high-risk superobese bariatric patients: a computed tomography-based analysis. *Surgery for Obesity and Related Diseases*. 2011;7(4):480-485. doi:10.1016/j.soard.2010.09.026

24. Yamaoka Y, Yamaguchi T, Kinugasa Y, et al. Mesorectal fat area as a useful predictor of the difficulty of robotic-assisted laparoscopic total mesorectal excision for rectal cancer. *Surg Endosc*. 2019;33(2):557-566. doi:10.1007/s00464-018-6331-9

25. McKechnie T, Ramji K, Kruse C, et al. Posterior mesorectal thickness as a predictor of increased operative time in rectal cancer surgery: a retrospective cohort study. *Surg Endosc*. 2022;36(5):3520-3532. doi:10.1007/s00464-021-08674-w

26. Ri M, Aikou S, Seto Y. Obesity as a surgical risk factor. *Ann Gastroenterol Surg*. 2018;2(1):13-21. doi:10.1002/ags3.12049

27. Wahl TS, Patel FC, Goss LE, Chu DI, Grams J, Morris MS. The obese colorectal surgery patient: Surgical site infection and outcomes. *Dis Colon Rectum*. 2018;61(8):938-945. doi:10.1097/DCR.0000000000001085

28. Geiger TM, Muldoon R. Complications following colon rectal surgery in the obese patient. *Clin Colon Rectal Surg*. 2011;24(4):274-282. doi:10.1055/s-0031-1295692

29. Chalfin HJ, Lee SB, Jeong BC, et al. Obesity and Long-Term Survival after Radical Prostatectomy. *Journal of Urology*. 2014;192(4):1100-1104. doi:10.1016/j.juro.2014.04.086

30. Kamineni A, Anderson ML, White E, et al. Body mass index, tumor characteristics, and prognosis following diagnosis of early-stage breast cancer in a mammographically screened population. *Cancer Causes & Control*. 2013;24(2):305-312. doi:10.1007/s10552-012-0115-7

31. Garneau P, Glazer S, Jackson T, et al. Guidelines for Canadian bariatric surgical and medical centres: a statement from the Canadian Association of Bariatric Physicians and Surgeons. *Canadian Journal of Surgery*. 2022;65(2):E170-E177. doi:10.1503/cjs.020719

32. Mechanick JI, Youdim A, Jones DB, et al. Clinical Practice Guidelines for the Perioperative Nutritional, Metabolic, and Nonsurgical Support of the Bariatric Surgery Patient—2013 Update: Cosponsored by American Association of Clinical Endocrinologists, The Obesity Society, and American Society for Metabolic &amp; Bariatric Surgery. *Surgery for Obesity and Related Diseases*. 2013;9(2):159-191. doi:10.1016/j.soard.2012.12.010

33. Ross LJ, Wallin S, Osland EJ, Memon MA. Commercial Very Low Energy Meal Replacements for Preoperative Weight Loss in Obese Patients: a Systematic Review. *Obes Surg*. 2016;26(6):1343-1351. doi:10.1007/s11695-016-2167-3

34. Atkinson RL. Very Low-Calorie Diets. *JAMA: The Journal of the American Medical Association*. 1993;270(8):967. doi:10.1001/jama.1993.03510080071034

35. Mulholland Y, Nicokavoura E, Broom J, Rolland C. Very-low-energy diets and morbidity: a systematic review of longer-term evidence. *British Journal of Nutrition*. 2012;108(5):832-851. doi:10.1017/S0007114512001924

36. van Nieuwenhove Y, Dambrauskas Z, Campillo-Soto A, et al. Preoperative very low-calorie diet and operative outcome after laparoscopic gastric bypass: A randomized multicenter study. *Archives of Surgery*. 2011;146(11):1300-1305. doi:10.1001/archsurg.2011.273

37. Liljensøe A, Laursen JO, Bliddal H, Søballe K, Mechlenburg I. Weight Loss Intervention Before Total Knee Replacement: A 12-Month Randomized Controlled Trial. *Scandinavian Journal of Surgery*. 2021;110(1):3-12. doi:10.1177/1457496919883812

38. Kip P, Trocha KM, Tao M, et al. Insights From a Short-Term Protein–Calorie Restriction Exploratory Trial in Elective Carotid Endarterectomy Patients. *Vasc Endovascular Surg*. 2019;53(6):470-476. doi:10.1177/1538574419856453

39. Barth RJ, Mills JB, Suriawinata AA, et al. Short-term Preoperative Diet Decreases Bleeding After Partial Hepatectomy. *Ann Surg*. 2019;269(1):48-52. doi:10.1097/SLA.0000000000002709

40. Burnand KM, Lahiri RP, Burr N, Jansen van Rensburg L, Lewis MPN. A randomised, single blinded trial, assessing the effect of a two week preoperative very low calorie diet on laparoscopic cholecystectomy in obese patients. *HPB*. 2016;18(5):456-461. doi:10.1016/j.hpb.2016.01.545

41. Ard JD, Lewis KH, Rothberg A, et al. Effectiveness of a Total Meal Replacement Program (OPTIFAST Program) on Weight Loss: Results from the OPTIWIN Study. *Obesity*. 2019;27(1):22-29. doi:10.1002/oby.22303

42. Griffin SB, Ross LJ, Burstow MJ, Desbrow B, Palmer MA. Efficacy of a dietitian-led very low calorie diet (VLCD) based model of care to facilitate weight loss for obese patients prior to elective, non-bariatric surgery. *Journal of Human Nutrition and Dietetics*. 2021;34(1):188-198. doi:10.1111/jhn.12819

43. Sousa-Santos AR, Amaral TF. Differences in handgrip strength protocols to identify sarcopenia and frailty - a systematic review. *BMC Geriatr*. 2017;17(1):238. doi:10.1186/s12877-017-0625-y

44. Deiro G, de Pastena M, Paiella S, et al. Assessment of difficulty in laparoscopic distal pancreatectomy: A modification of the Japanese difficulty scoring system – A single-center high-volume experience. *J Hepatobiliary Pancreat Sci*. 2021;28(9):770-777. doi:10.1002/jhbp.1010

45. Vannucci M, Laracca GG, Mercantini P, et al. Statistical models to preoperatively predict operative difficulty in laparoscopic cholecystectomy: A systematic review. *Surgery (United States)*. 2022;171(5):1158-1167. doi:10.1016/j.surg.2021.10.001

46. Raphael IJ, Parmar M, Mehrganpour N, Sharkey PF, Parvizi J. Obesity and operative time in primary total joint arthroplasty. *J Knee Surg*. 2013;26(2):95-99. doi:10.1055/s-0033-1333663

47. van Nieuwenhove Y, Dambrauskas Z, Campillo-Soto A, et al. *Preoperative Very Low-Calorie Diet and Operative Outcome After Laparoscopic Gastric Bypass A Randomized Multicenter Study*.

48. Grevitt M, Khazim R, Webb J, Mulholland R, Shepperd J. *THE SHORT FORM-36 HEALTH SURVEY QUESTIONNAIRE IN SPINE SURGERY*. Vol 79.; 1997.

49. Bernklev T, Jahnsen J, Lygren I, Henriksen M, Vatn M, Moum B. *Health-Related Quality of Life in Patients with Inflammatory Bowel Disease Measured with the Short Form-36: Psychometric Assessments and a Comparison with General Population Norms*. https://academic.oup.com/ibdjournal/article/11/10/909/4685935

50. Government of Canada. Differences in obesity rates between rural communities and urban cities in Canada. Government of Canada. Published November 5, 2020. Accessed October 24, 2022. https://health-infobase.canada.ca/datalab/canadian-risk-factor-atlas-obesity-blog.html?=undefined&wbdisable=true

51. Pessaux P, Msika S, Atalla D, Hay JM. *Risk Factors for Postoperative Infectious Complications in Noncolorectal Abdominal Surgery A Multivariate Analysis Based on a Prospective Multicenter Study of 4718 Patients*.

52. Davenport L, Johari Y, Klejn A, et al. Improving Compliance with Very Low Energy Diets (VLEDs) Prior to Bariatric Surgery—a Randomised Controlled Trial of Two Formulations. *Obes Surg*. 2019;29(9):2750-2757. doi:10.1007/s11695-019-03916-2

53. Schulz KF, Altman DG, Moher D. CONSORT 2010 Statement: updated guidelines for reporting parallel group randomised trials. *BMJ*. 2010;340(mar23 1):c332-c332. doi:10.1136/bmj.c332

54. Thabane L, Ma J, Chu R, et al. A tutorial on pilot studies: the what, why and how. *BMC Medical Research Methodology* . 2010;10. http://www.nsf.gov/pubs/2005/nsf0531/nsf0531_6.pdf

55. van Noorden R. Open Access: The True Cost of Science Publishing. *Nature*. 2013;495:426-429.

# BUDGET

| **Budget Details** | | | |
| --- | --- | --- | --- |
| 1. **Personnel** | **Hours** | **Salary** | **TOTAL** |
| McMaster Payroll Research Assistant 1: UNIFOR5555, Grade 6, Step 2, 35% Fringe Benefits | 20h/wk  52 wks  1,040 h | $33.75/h | $35,100.00 |
| McMaster Payroll Research Assistant 2: UNIFOR5555, Grade 6, Step 2, 35% Fringe Benefits | 10h/wk  52 wks  520 h | $33.75/h | $17,550.00 |
| Research Clerk (salary + 4% in-lieu of vacation) | 5h/wk  52 wks  260 h | $15.00/h | $4,056.00 |
| McMaster Biostatistician (35% Fringe Benefits) | 3h/wk  52 wks  156 h  (0.075 FTE) | Annual: $81,000  0.075 FTE | $6,075.00 |
| **Total Personnel** | | | **$62,781.00** |
| 1. **Supplies** | | | |
| Optifast® 900 program | | | $13,200.00 |
| Patient parking for non-routine clinical visits | | | $3,520.00 |
| Office supplies and printing | | | $1,000.00 |
| **Total Supplies** | | | **$17,720.00** |
| 1. **Services** | | | |
| Data Entry Services | | | $3,500.00 |
| Randomization Software | | | $2,500.00 |
| **Total Services** | | | **$6,000.00** |
| 1. **Knowledge Translation** | | | |
| Open Access Publication | | | $3,500.00 |
| Conference Presentation | | | $4,000.00 |
| **Total Knowledge Translation** | | | **$7,500.00** |
| **TOTAL AMOUNT** | | | **$94,001.00** |

## Budget justification

The total budget for this project is $94,001.00

Personnel

A Research Assistant is required to provide support for this project over the course of one year. The Research Assistant will be responsible for preparing study documents, aiding in Research Ethics Board applications, consenting patients, data collection, and liaising with the various trial stakeholders. The hourly wage of a Research Assistant at McMaster University is $25.00 +35% fringe benefits, which equates to $33.75 per hour. It is anticipated that they will work, on average, 20 hours per week for a total of 1 year on this project (20 hours/week x 52 weeks = 1,040 hours). As such, their compensation for the present project will be $33.75 x 1,040 hours = $31,500.00.

In addition, a second Research Assistant will be required to provide support for this project over the course of one year at the same cost as above, but at half the number of weekly hours. We will rely on this Research Assistant to obtain anthropometric measures for patients at their pre-very low energy diet (VLED) visit, post-VLED visit, and postoperative visit ($33.75 x 520 hours = $17,550.00).

A research clerk, paid at minimum wage ($15.00/h) for a temporary casual employee with an additional 4% wage in-lieu of vacation, will be employed to aid with input of paper data collection forms into electronic format to allow for data analysis. It is anticipated that they will work an average of 5 hours per week over the course of a year (5 hours/week x 52 weeks = 260 hours). As such, their compensation for the present project will be $15.00 x 260 hours + (4% x total) = ­$4,056.00.

The biostatistician is a staff statistician in the Department of Oncology at McMaster University. Under the supervision of the one of the study investigators, Dr. Sameer Parpia, they will be responsible for creating a randomization list, importing and formatting data for SAS/STATA software, cleaning the data, and completing the final analysis. It is estimated their services will be required for an average of 3 hours per week over the course of a year (0.075 full time equivalents (FTEs)). Their annual salary is $81,000. As such, the estimated cost for a year for their biostatistical services is 0.075 FTE x $81,000.00 = $6,075.00.

Supplies

All enrolled patients will adhere to the Optifast® 900 or Medimeal® program for three weeks. The weekly cost of the Optifast® 900 or Medimeal® program is approximately $100.00. The cost of the three-week program will be $300.00 ($100.00/wk x 3 weeks = $300.00) per patient and we will continue enrollment until 88 patients, half of which will be in the intervention arm (44 patients x $300.00/patient = $13,200.00).

Patients will be required to make to non-clinically scheduled in person visits for the purposes of the study. Following consent and enrollment, they will require baseline measures of height and weight, as well as symptoms, prior to commencing the study intervention/placebo. They will also require an in-person one-month postoperative follow-up for the purposes of the study that may not align with their scheduled follow up with their respective surgeon. Parking at St. Joseph’s Healthcare Hamilton or Hamilton Health Sciences is approximately $20.00 per visit. Given that each patient will have two visits (2 days x 88 patients x $20.00/patient/day) we will budget $3,520.00.

Patients will be provided with printed study information booklets, Optifast® 900 handouts, diet diaries, and a copy of their informed consent information. The SF-36 and symptom questionnaires will also be administered via paper forms. As such, $1,000.00 is anticipated for printing and associated office supply costs.

Services

Database preparation and entry of all study data will be organized under the supervision of the one of the study investigators, Dr. Sameer Parpia, Estimated cost is $3,500.00.

The randomization service provided by randomization.net costs $2,500.00.

Knowledge Translation

The final version of the manuscript from this project will be published in an Open Access journal in order to increase accessibility and aid in knowledge dissemination. The mean publishing fee for large Open Access journals (e.g., BioMed Central, PLoS) ranges from approximately $2,000.00 to $4,000.^55^

The findings generated from this pilot RCT will be presented at an international (e.g., Digestive Disease Week) and a national (e.g., Canadian Surgery Forum) conference. Registration, travel, and accommodations for each is approximately $2,000.00 ($2,000.00/conference x 2 conferences = $4,000.00.
