# Supplementary material for: Preoperative low-energy diets for patients with a body mass index >30 kg/m2 undergoing non-bariatric surgery: pilot feasibility randomized clinical trial and a systematic review and meta-analysis of efficacy data
Source: Br J Surg. 2026 Mar 13;113(5):znag023. doi: 10.1093/bjs/znag023 (PMC13155937; doi:10.1093/bjs/znag023)
Supplement: znag023_Supplementary_Data [file znag023_supplementary_data.zip › PREPARE_CONSORT_Abstract_Appendix2.doc]

CONSORT 2010 checklist of information to include when reporting a pilot or feasibility trial: abstract*

| Section/Topic | Checklist item | Reported on page No |
| --- | --- | --- |
| **Title** | Identification of study as randomized pilot trial | 1 |
| **Trial Design** | Description of pilot trial design (e.g., parallel, cluster) | 5 |
| **METHODS** | | |
| **Participants** | Eligibility criteria for participants and the settings where the pilot trial was conducted | 5 |
| **Interventions** | Interventions intended for each group | 5 |
| **Objective** | Specific objectives of the pilot trial | 5 |
| **Outcome** | Pre-specified assessment or measurement to address the pilot trial objective(s) | 5 |
| **Randomization** | How participants were allocated to interventions | N/A |
| **Blinding (masking)** | Whether or not participants, care givers, and those assessing the objectives were blinded to group assignment | N/A |
| **RESULTS** | | |
| **Numbers randomized** | Number of participants screened and randomized to each group for the pilot trial objective(s) | 5 |
| **Recruitment** | Trial status | 5 |
| **Numbers analysed** | Number of participants analyzed in each group for the pilot objective(s) | 5 |
| **Outcome** | Results for the pilot objective(s); including any expression of uncertainty | 5 |
| **Harms** | Important adverse events or side-effects | 5 |
| **Conclusions** | General interpretation of the results of pilot trial and their implications for the future definitive trial | 5 |
| **Trial Registration** | Registration number for pilot trial and name of trial register | 5 |
| **Funding** | Source of funding for pilot trial | 2 |
